# Viable Neuronopathic Gaucher Disease Model in Medaka (*Oryzias latipes*) Displays Axonal Accumulation of Alpha-Synuclein

**DOI:** 10.1371/journal.pgen.1005065

**Published:** 2015-04-02

**Authors:** Norihito Uemura, Masato Koike, Satoshi Ansai, Masato Kinoshita, Tomoko Ishikawa-Fujiwara, Hideaki Matsui, Kiyoshi Naruse, Naoaki Sakamoto, Yasuo Uchiyama, Takeshi Todo, Shunichi Takeda, Hodaka Yamakado, Ryosuke Takahashi

**Affiliations:** 1 Department of Neurology, Kyoto University Graduate School of Medicine, Kyoto, Japan; 2 Department of Cell Biology and Neuroscience, Juntendo University Graduate School of Medicine, Tokyo, Japan; 3 Division of Applied Biosciences, Kyoto University Graduate School of Agriculture, Kyoto, Japan; 4 Department of Radiation Biology and Medical Genetics, Osaka University Graduate School of Medicine, Suita, Japan; 5 National Institute for Basic Biology, Laboratory of Bioresources, Okazaki, Japan; 6 Department of Mathematical and Life Sciences, Hiroshima University Graduate School of Science, Higashi-Hiroshima, Japan; 7 Department of Radiation Genetics, Kyoto University Graduate School of Medicine, Kyoto, Japan; 8 Core Research for Evolutional Science and Technology, Japan Science and Technology Agency, Kawaguchi, Japan; Stanford University School of Medicine, UNITED STATES

## Abstract

Homozygous mutations in the *glucocerebrosidase* (*GBA*) gene result in Gaucher disease (GD), the most common lysosomal storage disease. Recent genetic studies have revealed that *GBA* mutations confer a strong risk for sporadic Parkinson’s disease (PD). To investigate how *GBA* mutations cause PD, we generated *GBA* nonsense mutant (*GBA*-/-) medaka that are completely deficient in glucocerebrosidase (GCase) activity. In contrast to the perinatal death in humans and mice lacking GCase activity, *GBA*-/- medaka survived for months, enabling analysis of the pathological progression. *GBA*-/- medaka displayed the pathological phenotypes resembling human neuronopathic GD including infiltration of Gaucher cell-like cells into the brains, progressive neuronal loss, and microgliosis. Detailed pathological findings represented lysosomal abnormalities in neurons and alpha-synuclein (α-syn) accumulation in axonal swellings containing autophagosomes. Unexpectedly, disruption of *α-syn* did not improve the life span, formation of axonal swellings, neuronal loss, or neuroinflammation in *GBA*-/- medaka. Taken together, the present study revealed *GBA*-/- medaka as a novel neuronopathic GD model, the pahological mechanisms of α-syn accumulation caused by GCase deficiency, and the minimal contribution of α-syn to the pathogenesis of neuronopathic GD.

## Author Summary

Parkinson’s disease (PD) is a neurodegenerative disease characterized by intraneuronal accumulation of alpha-synuclein (α-syn) called Lewy bodies and Lewy neurites. Recent genetic studies have revealed that mutations in *glucocerebrosidase* (*GBA*), a causative gene of Gaucher disease (GD), are a strong risk for PD. However, its pathological mechanisms leading to PD remain largely unknown. Here, we generated *GBA* nonsense mutant (*GBA* ^-/-^) medaka which survive long enough for pathological analysis of disease progression. These mutant medaka display not only the phenotypes resembling human neuronopathic GD but also axonal accumulation of α-syn accompanied by impairment of the autophagy-lysosome pathway. Furthermore, the present study demonstrates this α-syn accumulation has negligible contribution to the pathogenesis of neuronopathic GD in medaka. *GBA* ^-/-^ medaka represent a valuable model for exploring the pathological mechanisms of PD with *GBA* mutations as well as neuronopathic GD, and our findings have important implications for the association of *GBA* mutations with PD.

## Introduction

Gaucher disease (GD) is the most common lysosomal storage disease and is caused by homozygous mutations in *glucocerebrosidase* (*GBA*). Mutations in *GBA* lead to decreased enzymatic activity of glucocerebrosidase (GCase) and result in the accumulation of its substrates, glucocerebroside and glucosylsphingosine[1,2]. GD is classically divided into three subtypes: a non-neuronopathic form (type 1), an acute neuronopathic form (type 2), and a subacute neuronopathic form (type 3). Visceral manifestations of all forms are characterized by hepatosplenomegaly, cytopenia, and skeletal disease. Pathologically, the accumulation of lipid-laden macrophages, called Gaucher cells, are observed in the affected organs. Neurological manifestations of neuronopathic forms include brainstem dysfunction, intellectual disability, seizures, and myoclonic movement. Pathological features of neuronopathic forms are neuronal loss, astrogliosis, microgliosis, and perivascular accumulation of Gaucher cells[3]. The most severe neuronopathic form, called the perinatal lethal type, has also been reported[4]. Common presentations of patients with the perinatal lethal type are hydrops fetalis and congenital ichthyosis. Almost no residual GCase enzymatic activity is found in these cases. Because currently available therapies are ineffective for neurological manifestations, a strong demand exists for elucidation of the pathological mechanisms and the development of novel therapies.

Parkinson’s disease (PD) is the most common neurodegenerative movement disorder. *GBA* has recently drawn considerable attention because heterozygous mutations in this gene confer a high risk for sporadic PD[5,6]. In addition, patients with type 1 GD also have an increased life-time risk of developing PD[7]. PD patients carrying *GBA* mutations show intraneuronal accumulation of alpha-synuclein (α-syn) called Lewy bodies and Lewy neurites, which are the pathological hallmarks of sporadic PD[3]. Several cellular, animal, and postmortem studies have indicated an association between *GBA* mutations and α-syn accumulation. For example, deficiency in GCase enzymatic activity causes lysosomal dysfunction and α-syn accumulation[8,9,10,11,12]. Increased α-syn in turn creates a vicious cycle by inhibiting the trafficking of GCase to lysosomes, thus leading to decreased GCase activity in lysosomes[9]. Consistent with this notion, mouse models overexpressing α-syn and postmortem tissue from patients with PD show reduced GCase activity in the brains[13,14,15]. Although several hypotheses have been proposed, further mechanisms of how *GBA* mutations contribute to the development of PD remain elusive.

Medaka (*Oryzias latipes*) are a versatile vertebrate animal model for disease research. These fish are easy to handle, have a relatively short generation time (2–3 months), produce a large number of progeny per generation, and have several inbred strains[16]. Importantly, medaka have an advantage as an animal model of PD due to endogenous α-syn in contrast to invertebrate models that lack α-syn. Moreover, several genetic manipulations can be performed in medaka in addition to established transgenic techniques[17,18,19,20,21]. So far, we have reported genetic PD models of medaka that develop locomotor dysfunction accompanied by the selective loss of dopaminergic and noradrenergic neurons[22,23]. Considering these lines of evidence, medaka have the potential to be a new animal model of PD.

Here, we generated *GBA* nonsense mutant medaka and found that homozygous *GBA* nonsense mutant (*GBA* ^-/-^) medaka are a viable neuronopathic GD model. *GBA* ^-/-^ medaka developed remarkable α-syn accumulation in the brains and thus provide novel insights into the association of *GBA* mutations with α-syn accumulation. Furthermore, we revealed minimal contribution of endogenous α-syn to the pathogenesis of neuronopathic GD in medaka.

## Results

### Generation of *GBA* nonsense mutant medaka

**Fig 1:**
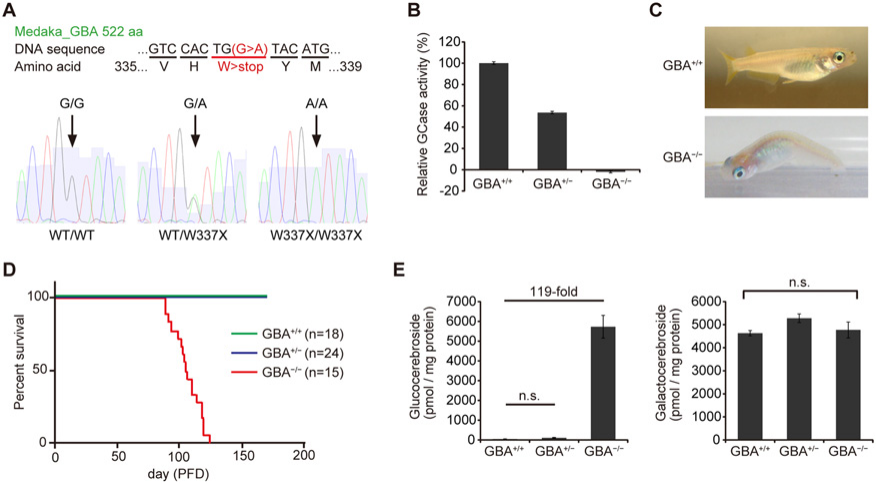
Generation of *GBA* nonsense mutant medaka. (A) Upper panel: DNA and amino acid sequences of *GBA* nonsense mutant medaka. Lower panels: Sequence data for each genotype. A = green, T = red, G = black, and C = blue. (B) Relative GCase activity in medaka brains (n = 10). (C) Abnormal posture (‘bent spine’) in *GBA* ^-/-^ medaka at 3 months. (D) Survival curves for each genotype. PFD, post-fertilization day. (E) Quantification of glucocerebroside and galactocerebroside in medaka brains at 3 months with SFC/MS/MS (n = 3–4). For all analyses, data are the mean ± standard error of the mean (SEM). n.s. means not significant.

We generated *GBA* nonsense mutant medaka to investigate the mechanisms by which *GBA* mutation leads to PD. To identify medaka *GBA* orthologs, we searched the medaka genome database (http://www.ensembl.org/Oryzias_latipes/Info/Index) with the basic local alignment search tool and found only one ortholog of human *GBA*. We cloned the medaka *GBA* with reverse transcription-polymerase chain reaction (RT-PCR) and rapid amplification of cDNA ends and found that this gene has 11 exons encoding a protein of 522 amino acids. The amino acid sequence of medaka *GBA* showed 53% homology to that of human *GBA* (S1 Fig). Next, we screened a targeting-induced local lesions in genome (TILLING) library for medaka *GBA* using a high-resolution melting assay[17,24]. We identified a nonsense mutant (W337X) and generated the nonsense mutant medaka by *in vitro* fertilization (Fig. 1A). We examined GCase activity in the brains of *GBA* mutants after crossing with heterozygous mutants. *GBA* ^*W337X/W337X*^ (*GBA* ^-/-^) medaka showed complete deficiency in GCase activity, and *GBA* ^*WT/W337X*^ (*GBA* ^+/-^) medaka showed a decrease in GCase activity of about 50% compared to wild-type (*GBA* ^*+/+*^) medaka (Fig. 1B). Although humans and mice lacking GCase activity die soon after birth[4,25,26], *GBA* ^-/-^ medaka survived for more than 3 months, enabling us to analyze the pathological progression (Fig. 1D). *GBA* ^-/-^ medaka showed abnormal rotating swimming movement at 2 months (S1, S2 Movie) and the abnormal appearance of a bent spine at 3 months (Fig. 1C). High levels of glucocerebroside accumulated in the brains of *GBA* ^-/-^ medaka (Fig. 1E), whereas the amount of galactocerebroside, an isomer of glucocerebroside, was not changed. Glucocerebroside with C18 fatty acids was the most dominant type in the brains of *GBA* ^-/-^ medaka (S2 Fig), an observation that is consistent with the neuronopathic GD mouse model[27].

### *GBA* ^-/-^ medaka showed neuronopathic GD-like pathology

**Fig 2:**
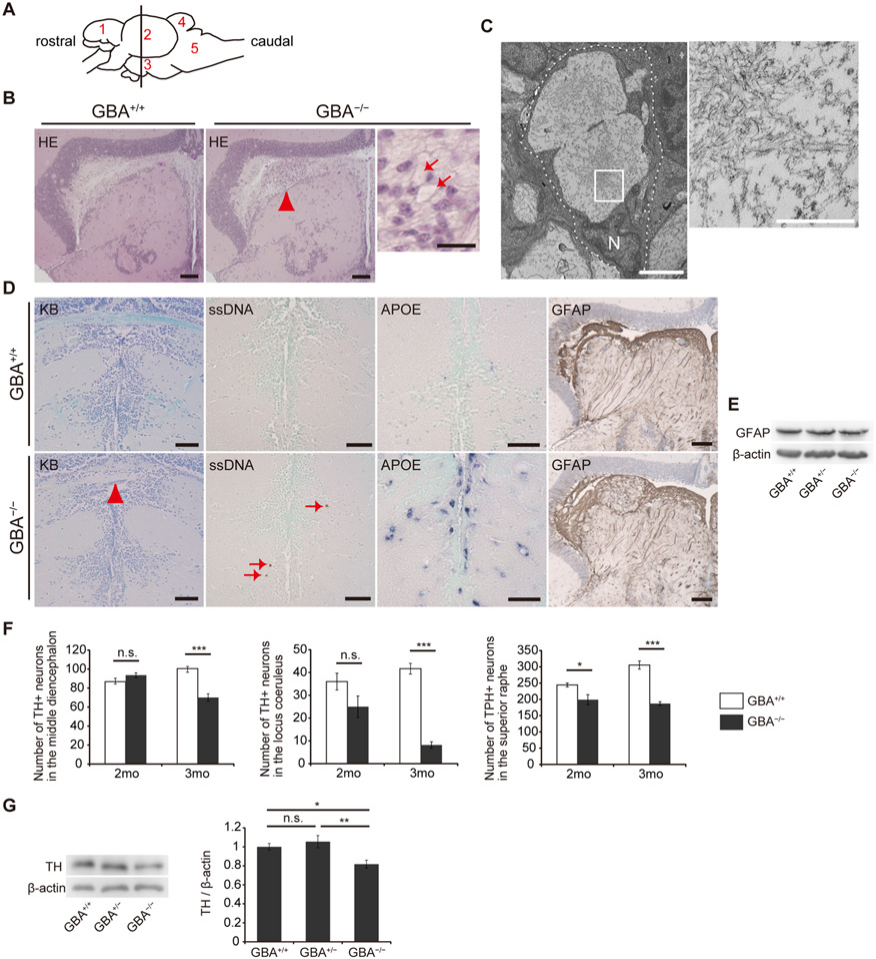
Pathological analyses of *GBA* ^-/-^ medaka. (A) Schematic of a lateral view of the medaka brain. Each number signifies a part of the brain. 1: telencephalon, 2: optic tectum, 3: diencephalon, 4: cerebellum, 5: medulla oblongata. The brain sections used for pathological analyses in the present study are illustrated by the vertical line. (B) Hematoxylin and eosin staining. Abnormal cells observed in the periventricular gray zone of the optic tectum (arrowhead) in *GBA* ^-/-^ medaka at 3 months. Scale bars, 50 μm. Right panel: High-magnification image showing abnormal cells with large vacuoles (arrows). Scale bar, 5 μm. (C) Transmission electron micrographs showing abnormal macrophage-like cells. Left panel: A whole-cell image of an abnormal macrophage-like cell. Dashed lines outline a whole abnormal cell. N, Nucleus. Scale bar, 2 μm. Right panel: High-magnification image of filamentous structures in vacuoles. Scale bar, 500 nm. (D) Klüver-Barrera (KB) staining, ssDNA immunohistochemistry, *APOE* in situ hybridization, and GFAP immunohistochemistry in the diencephalon. Commissura posterior with decreased Luxol fast blue staining intensity (arrowhead) and ssDNA-positive dead cells (arrows) in *GBA* ^-/-^ medaka. *APOE* in situ hybridization revealed proliferating activated microglia in *GBA* ^-/-^ medaka. The staining intensity and area of GFAP were not changed in *GBA* ^-/-^ medaka. Scale bars, 50 μm. (E) Western blot analysis of GFAP and β-actin. The expression level of GFAP was not changed among genotypes. (F) Number of TH-positive neurons in the middle diencephalon, number of TH-positive neurons in the locus coeruleus, and number of TPH-positive neurons in the superior raphe at 2 and 3 months. In *GBA* ^-/-^ medaka, progressive neuronal loss was observed in all types of neurons (n = 4–6, *p < 0.05, ***p < 0.001). (G) Western blot analysis of TH and β-actin (n = 7, *p < 0.05, **p < 0.01). For all analyses, data are the mean ± SEM.

We performed pathological analyses of *GBA* ^-/-^ medaka. Patients with GD show Periodic acid-Schiff-positive Gaucher cells in affected visceral organs such as the liver, spleen, and bone marrow, whereas *GBA* ^-/-^ medaka showed abnormal Periodic acid-Schiff-positive cells in the spleen and kidney, but not in the liver, at 3 months (S3 Fig). Next, we examined the brains of *GBA* ^-/-^ medaka and found abnormal cells with large vacuoles mainly in the periventricular gray zone of the optic tectum (Fig. 2A, B). Transmission electron microscopy revealed that these cells were macrophage-like and had large vacuoles containing filamentous structures (Fig. 2C). Similar filamentous structures are observed in Gaucher cells of patients with GD and mouse models of GD[26,28,29]. The staining intensity with Luxol fast blue was decreased, and single-stranded DNA (ssDNA)-positive cells were observed in *GBA* ^-/-^ medaka (Fig. 2D), indicating myelin loss and cell death, respectively. In situ hybridization for *apolipoprotein E* (*APOE*), a microglial marker in teleost fish[30], revealed proliferating activated microglia in *GBA* ^-/-^ medaka (Fig. 2D). Teleost fish have glial fibrillary acidic protein (GFAP)-expressing radial glial cells (or ependymoglial cells) instead of astrocytes as in mammals[31]. Humans and mice with neuronopathic GD show astrogliosis in their brains[3,32], whereas neither proliferation of GFAP-positive radial glial cells nor elevated levels of GFAP were observed in *GBA* ^-/-^ medaka (Fig. 2D, E). To investigate the type of neuronal cells that die in *GBA* ^-/-^ medaka, we counted the numbers of tyrosine hydroxylase (TH)-positive dopaminergic neurons in the middle diencephalon, which corresponds to the human substantia nigra[33], TH-positive noradrenergic neurons in the locus coeruleus, and tryptophan hydroxylase (TPH)-positive serotonergic neurons in the superior raphe at 2 and 3 months. *GBA* ^-/-^ medaka showed progressive cell loss of all these neurons (Fig. 2F). Consistent with these findings, the amount of TH was decreased in *GBA* ^-/-^ medaka (Fig. 2G). Collectively, *GBA* ^-/-^ medaka exhibited neuronopathic GD-like pathology including progressive and non-selective neuronal loss.

### *GBA* ^-/-^ medaka developed α-syn accumulation in axonal swellings containing autophagosomes

Medaka express α-syn, which is a protein consisting of 127 amino acids. To investigate α-syn pathology in *GBA* ^-/-^ medaka, we created a medaka α-syn antibody against the epitope of amino acids 90 to 104 (S4A Fig). Medaka also express β-synuclein, γ-synuclein-a, and γ-synuclein-b in addition to α-syn, but these other synucleins do not have amino acid sequences homologous to the epitope of the medaka α-syn antibody (S4B Fig). Next, we generated *α-syn* deletion mutant medaka using TALENs. Deletion of 11 bases near the start codon in *α-syn* resulted in a frame shift mutation (S4C Fig). Wild-type, heterozygous, and homozygous *α-syn* deletion mutant (*α-syn* ^*+/+*^, *α-syn* ^+/-^, and *α-syn* ^-/-^, respectively) medaka could be distinguished with PCR analysis of *α-syn* (S4D Fig). RT-PCR analysis of *α-syn* mRNA revealed that *α-syn* ^-/-^ medaka did not express intact *α-syn* mRNA (S4E Fig). Western blot analysis with the medaka α-syn antibody revealed a 14-kDa band, which was specifically found in *α-syn* ^*+/+*^ medaka (S4F Fig). The authenticity of the antibody was confirmed by the lack of immunostaining with the medaka α-syn antibody in the brains of *α-syn* ^-/-^ medaka (S4G Fig). Consistent with the findings in mammals, medaka α-syn was mainly found presynaptically with immunoelectron microscopy (S4H Fig).

**Fig 3:**
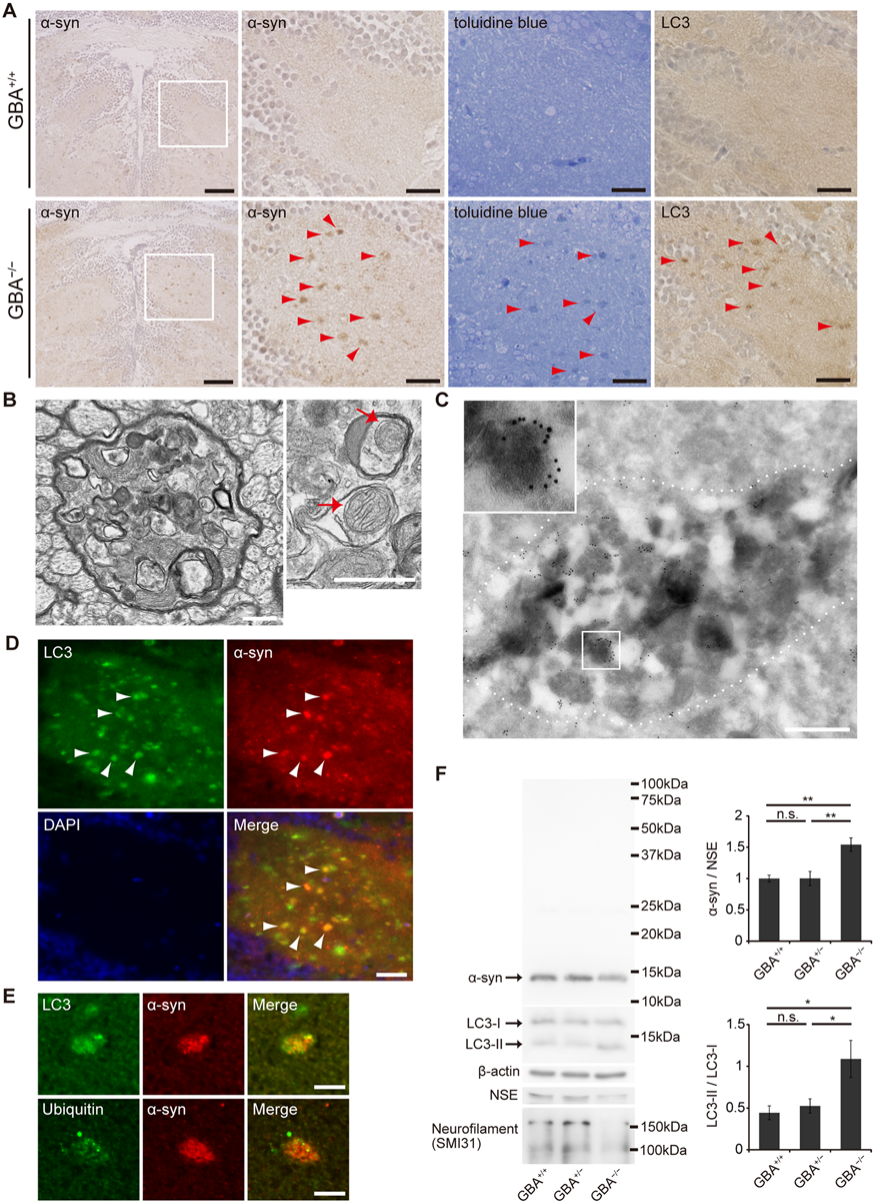
Axonal swellings with α-syn accumulation in *GBA* ^-/-^ medaka. (A) α-syn and LC3 immunohistochemistry, and toluidine blue staining in the diencephalon at 3 months. Left panels: α-syn accumulations were observed in *GBA* ^-/-^ medaka. Scale bars, 50 μm. Other panels: outlined area of left panels. The distribution of α-syn accumulations was similar to that of abnormal structures observed with toluidine blue staining and LC3 accumulations (arrowheads). Scale bars, 20 μm. (B) Transmission electron micrographs of axonal swellings in *GBA* ^-/-^ medaka. Left panel: a swelling of a myelinated axon containing vacuoles and electron-dense bodies. Right panel: vacuoles in an axonal swelling containing mitochondria (arrows). Scale bars, 500 nm. (C) Immunoelectron micrograph of an axonal swelling in a *GBA* ^-/-^ medaka with immunogold-labeled α-syn. Dashed lines outline an axonal swelling containing vacuoles and electron-dense bodies. The boxed area is enlarged in the inset. Scale bar, 500 nm. (D) Double immunostaining for LC3 (green) and α-syn (red) in *GBA* ^-/-^ medaka. Nuclei were visualized with DAPI (blue). α-syn accumulations colocalized with LC3 accumulations (arrowheads). Scale bar, 20 μm. (E) Conforcal microscope images of α-syn-positive axonal swellings. Upper panels: Double immunostaining for LC3 (green) and α-syn (red). A considerable portion of the α-syn signals colocalized with LC3 signals. Lower panels: Double immunostaining for ubiquitin (green) and α-syn (red). Ubiquitin colocalized with an α-syn accumulation. Scale bars, 5 μm. (F) Western blot analysis of α-syn, LC3, β-actin, NSE, and neurofilament at 3 months (n = 6–7, *p < 0.05, **p < 0.01). For all analyses, data are the mean ± SEM.

Then, we performed immunohistochemical analysis and found abundant α-syn accumulation in the brains of *GBA* ^-/-^ medaka at 3 months (Fig. 3A). α-syn accumulation was also observed at 2 months, but not at 1 month (S5 Fig). Abnormal structures observed with toluidine blue staining were similar to α-syn accumulations in size and distribution (Fig. 3A). Transmission electron microscopy revealed numerous axonal swellings containing vacuoles and other various materials in the brains of *GBA* ^-/-^ medaka (Figs. 3B, S6A). A considerable number of these vacuoles had double membranes and sometimes contained mitochondria, suggesting that these vacuoles were autophagosomes. Consistent with this, LC3, an autophagosomal marker, accumulated in the brains of *GBA* ^-/-^ medaka (Fig. 3A). Moreover, immunogold-labeled α-syn was detected in axonal swellings with immunoelectron microscopy (Fig. 3C). Conventional and confocal double immunofluorescence microscopy revealed that accumulated α-syn colocalized with LC3 accumulations (Fig. 3D), and a considerable portion of the α-syn signals in axonal swellings colocalized with LC3 signals (Figs. 3E, S6B). In addition to these findings, ubiquitin also accumulated in α-syn-positive axonal swellings (Figs. 3E, S6B). To examine the α-syn expression level and solubility, we performed sequential biochemical fractionation assays. The total amount of α-syn in the Triton-soluble fraction was not increased in *GBA* ^-/-^ medaka. However, when adjusted for the amount of neuron-specific enolase (NSE) and considering the robust neuronal loss in *GBA* ^-/-^ medaka, α-syn was significantly increased in *GBA* ^-/-^ medaka (Fig. 3F). The decreased amount of neurofilament also reflected the neuronal loss in *GBA* ^-/-^ medaka (Fig. 3F). No apparent α-syn band was detected in the Triton-insoluble, SDS-soluble fraction in all genotypes. In agreement with the accumulation of autophagosomes in axonal swellings, the ratio of LC3-II to LC3-I was increased in *GBA* ^-/-^ medaka (Fig. 3F).

Meanwhile, we also investigated the phenotypes of *GBA* ^+/-^ medaka because heterozygous mutations in *GBA* are a strong risk for PD[5,6]. However, *GBA* ^+/-^ medaka even at 12 months did not show any apparent abnormal phenotypes including α-syn pathology, the numbers of TH-positive neurons, swimming movement, or the amounts of several neurotransmitters (S7A–S7E Fig).

### Impairment of the autophagy-lysosome pathway in neurons of *GBA* ^-/-^ medaka

**Fig 4:**
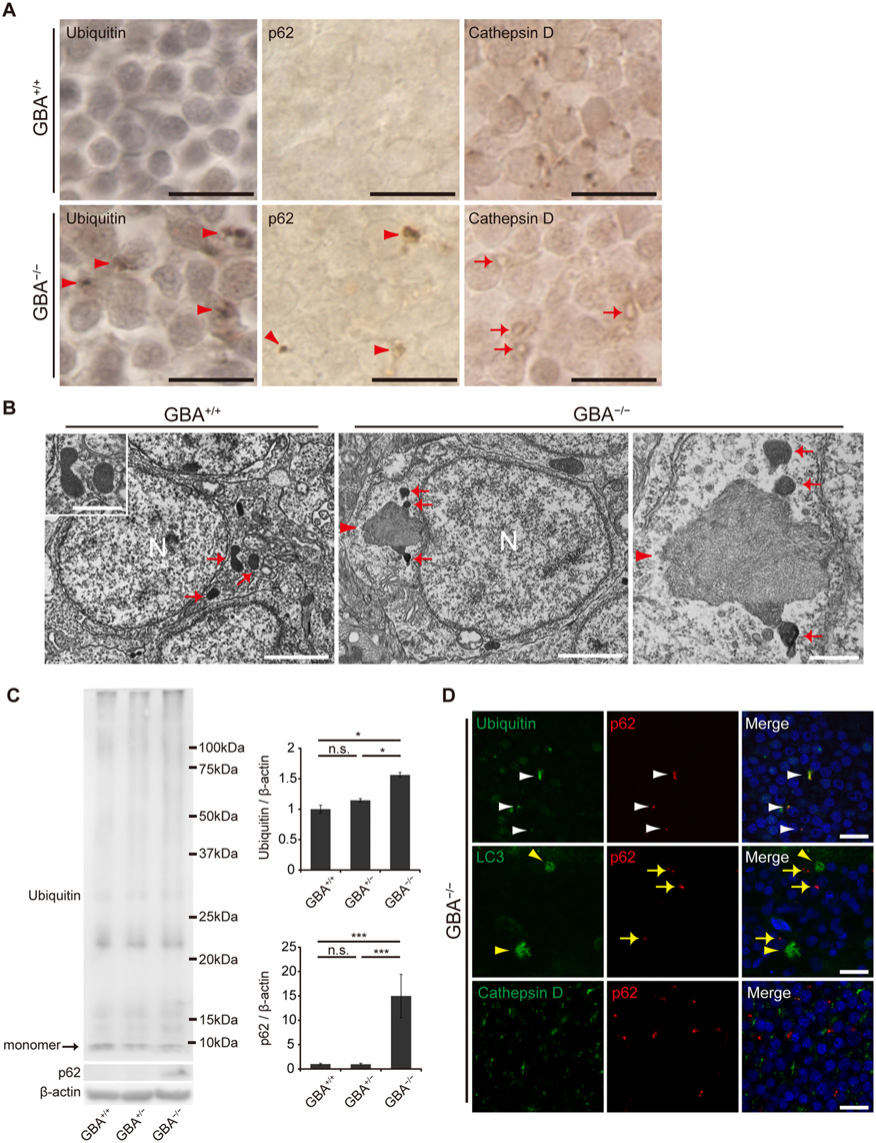
Impairment of the autophagy-lysosome pathway in *GBA* ^-/-^ medaka. (A) Ubiquitin, p62, and Cathepsin D immunohistochemistry in the neuronal layer of the optic tectum at 3 months. Almost all of the cells in these figures are neurons. Ubiquitin-positive and p62-positive aggregates were observed in the perikarya of neurons in *GBA* ^-/-^ medaka at 3 months (arrowheads). Morphologically abnormal organelles with decreased Cathepsin D staining intensity were observed in *GBA* ^-/-^ medaka (arrows). Scale bars, 10 μm. (B) Transmission electron micrographs of neurons. Left panel: Neuron of a *GBA* ^*+/+*^ medaka. Electron-dense organelles (arrows) are likely lysosomes. N, Nucleus. Scale bar, 2 μm. The inset shows a high-magnification image of electron-dense organelles. Scale bar, 500 nm. Middle panel: Neuron of a *GBA* ^-/-^ medaka. An aggregate containing filamentous structures (arrowhead) is continuous with an electron-dense organelle. N, Nucleus. Scale bar, 2 μm. Right panel: High-magnification image of an aggregate of filamentous structures (arrowhead) and electron-dense organelles (arrows) in a *GBA* ^-/-^ medaka. Scale bar, 500 nm. (C) Western blot analysis of ubiquitin, p62, and β-actin (n = 4–7, *p < 0.05, ***p < 0.001). (D) Conforcal microscope images of *GBA* ^-/-^ medaka. Upper panels: ubiquitin (green) and p62 (red). p62-positive aggregates colocalized with ubiquitin-positive aggregates (white arrowheads). Middle panels: LC3 (green) and p62 (red). p62-positive aggregates (yellow arrows) did not colocalize with LC3 accumulations (yellow arrowheads). Lower panels: Cathepsin D (green) and p62 (red). p62-positive aggregates did not colocalize with Cathepsin D-positive organelles. Nuclei were visualized with DAPI (blue). Scale bars, 10 μm. For all analyses, data are the mean ± SEM.

Previous studies of GD mouse models reported that p62/SQSTM1 (an autophagic substrate) accumulates in neurons and astrocytes[34], and the number of Cathepsin D-positive puncta is decreased in neurons[35]. These observations prompted us to examine the autophagy-lysosome pathway in *GBA* ^-/-^ medaka. Immunohistochemical analysis revealed that ubiquitin- and p62-positive aggregates were observed mainly in the perikarya of neurons (Figs. 4A, D, S8B). These aggregates were observed only in neurons and not in GFAP-positive radial glial cells or Lycopersicon Esculentum (Tomato) Lectin (LEL)-positive microglia (S8A Fig). LEL is an excellent teleost microglial marker[36]. Western blot analysis showed that the amounts of ubiquitin and p62 were increased in the brains of *GBA* ^-/-^ medaka (Fig. 4C). In contrast, these aggregates did not colocalize with LC3 accumulations or Cathepsin D-positive organelles (Figs. 4D, S8B). These organelles, which are putative lysosomes, showed decreased Cathepsin D staining intensity and abnormal morphology (Fig. 4A). Consistent with this, neurons that contained lysosome-like organelles filled with filamentous structures were observed with transmission electron microscopy (Fig. 4B).

### The phenotypes of *GBA* ^-/-^ medaka were rescued by transgenic expression of *GBA*

*GBA* nonsense mutant medaka have random point mutations in the genome at loci other than *GBA*. Therefore, we performed a rescue experiment to determine whether the abnormal phenotypes observed in *GBA* ^-/-^ medaka were really caused by *GBA* mutation. We created medaka *GBA*-expressing vectors driven by a medaka *growth-associated protein 43* (*GAP-43*) promoter (S9A Fig)[37]. *GAP-43* mRNA is expressed mainly in nervous system in medaka[37]. We established six medaka *GBA* transgenic lines (*Tg(GAP-43*:*GBA*)) using these vectors and crossed them with *GBA* nonsense mutant medaka. Each line of *GBA* ^-/-^ medaka with *GBA* transgene (*Tg(GAP-43*:*GBA);GBA* ^-/-^) showed GCase activity of various levels in the brains at 3 months (S9B Fig). All lines of *Tg(GAP-43*:*GBA);GBA* ^-/-^ medaka showed normal swimming movement at 2 months (S3 Movie). Also, the swimming distance was increased in *Tg(GAP-43*:*GBA)line3;GBA* ^-/-^ medaka (S9C Fig). Pathological analysis of the brains of *Tg(GAP-43*:*GBA)line3;GBA* ^-/-^ medaka including hematoxylin and eosin staining, in situ hybridization for *APOE*, and immunohistochemistry for p62 and α-syn revealed no apparent abnormality (S9D Fig). We concluded that the abnormal phenotypes observed in the brains of *GBA* ^-/-^ medaka were caused by the *GBA* mutation.

### Disruption of *α-syn* in *GBA* ^-/-^ medaka did not improve the phenotypes

Many studies have reported that accumulated α-syn caused by α-syn overexpression results in neurotoxicity[38]. Neuroinflammation is observed in the brains of patients with PD, and much evidence shows that neuroinflammation promotes disease progression[39]. Moreover, a recent study reported that oligomeric α-syn released from neurons activates inflammatory responses in microglia[40].

**Fig 5:**
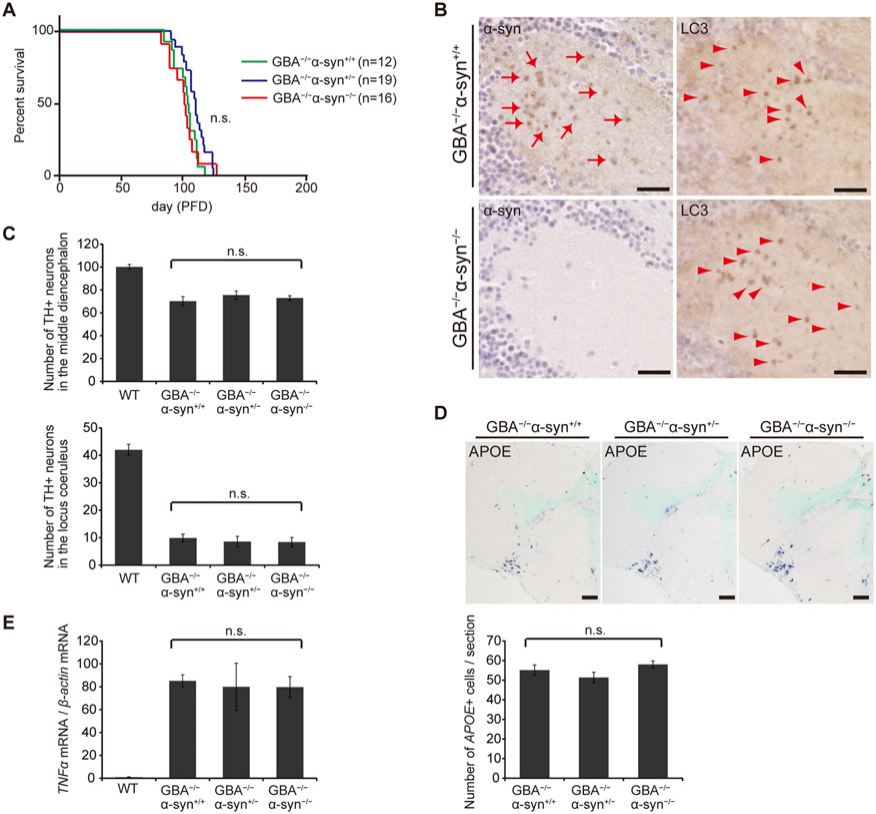
Disruption of *α-syn* did not change the pathological phenotypes in *GBA* ^-/-^ medaka. (A) Survival curves for each genotype. Life spans were not changed among genotypes. (B) α-syn and LC3 immunohistochemistry in the diencephalon. α-syn accumulations were observed only in *GBA* ^-/-^ *α-syn* ^*+/+*^ medaka (arrows). The number of LC3-positive puncta was not different between *GBA* ^-/-^ *α-syn* ^*+/+*^ and *GBA* ^-/-^ *α-syn* ^-/-^ medaka (arrowheads). Scale bars, 20 μm. (C) Number of TH-positive neurons in the middle diencephalon and the locus coeruleus at 3 months (n = 6). (D) Upper panel: *APOE* in situ hybridization in the optic tectum and the diencephalon. Lower panel: The number of *APOE*-positive cells per section (n = 9). Scale bars, 50 μm. (E) Quantification of *TNFα* mRNA levels normalized to *β-actin* mRNA in medaka brains with quantitative RT-PCR (n = 8). For all analyses, data are the mean ± SEM.

We examined the toxicity of α-syn in *GBA* ^-/-^ medaka by crossing *GBA* nonsense mutant medaka with *α-syn* deletion mutant medaka. *α-syn* ^-/-^ medaka showed no apparent abnormal phenotypes in their outer appearance, swimming movement, or life span like *α-syn*-disrupted mice[41]. The life spans of *GBA* ^-/-^ *α-syn* ^+/-^ and *GBA* ^-/-^ *α-syn* ^-/-^ medaka were not prolonged compared to that of *GBA* ^-/-^ *α-syn* ^*+/+*^ medaka (Fig. 5A). Moreover, the number of LC3-positive puncta was not changed in *GBA* ^-/-^ *α-syn* ^-/-^ medaka, indicating that α-syn was not primarily involved in formation of axonal swellings (Fig. 5B). The numbers of dopaminergic neurons in the middle diencephalon and noradrenergic neurons in the locus coeruleus were not changed in either *GBA* ^-/-^ *α-syn* ^+/-^ or *GBA* ^-/-^ *α-syn* ^-/-^ medaka (Fig. 5C). We also examined the extent of neuroinflammation with in situ hybridization for *APOE* and quantitative RT-PCR for *tumor necrosis factor* (*TNF*)*α* mRNA. The number of *APOE*-positive cells and the expression level of *TNFα* were not changed (Fig. 5D, E). Collectively, we found no evidence for α-syn involvement in the short life span, formation of axonal swellings, dopaminergic and noradrenergic neuronal loss, and neuroinflammation that are observed in *GBA* *-/-* medaka.

## Discussion

To date, several genetic animal models of neuronopathic GD including mouse, dog, and sheep have been reported[42]. *Gba* null mice die within 24 hours of birth due to permeability barrier defects in the skin[26,43]. Thus, conditional knockout K14-lnl/lnl mice with normal GCase activity in their skin were generated[27]. However, use of these mice is limited because they survive only 2 weeks after birth. Mice harboring a *Gba* missense mutation combined with prosaposin- or saposin C-deficient mice, another neuronopathic GD model, also show neuronopathic abnormalities[29,34] and have the advantage of longer survival than K14-lnl/lnl mice. However, the relevance of these mice to neuronopathic GD is controversial. The present study revealed that *GBA* ^-/-^ medaka survive long enough for pathological analysis of disease progression. As an example, *GBA* ^-/-^ medaka showed α-syn accumulation at 2 months, not at 1 month after fertilization. Considering these observations, *GBA* ^-/-^ medaka are useful as a viable neuronopathic GD model with endogenous α-syn accumulation.

*GBA* ^-/-^ medaka showed several phenotypes different from those of mammalian neuronopathic GD model. Firstly, the skin of *GBA* ^-/-^ medaka looks intact whereas severe skin lesion is observed in patients with perinatal lethal type GD and *Gba* null mice[4,43]. Secondly, *GBA* ^-/-^ medaka exhibited PAS-positive abnormal cells in spleen and kidney which presumably correspond to human Gaucher cells. In patients with GD, Gaucher cells are not found in kidney, but in liver, spleen and bone marrow. These differences could be explained by the fact that in adult teleost fish kidney has hematopoietic function instead of mammalian bone marrow[44]. Lastly, the proliferation of GFAP-positive radial glial cells was not observed in *GBA* ^-/-^ medaka whereas astrogliosis is observed in humans and mice with neuronopathic GD. It was reported that the reaction of GFAP-positive radial glial cells to inflammation is different from that of mammalian astrocytes[31].

**Fig 6:**
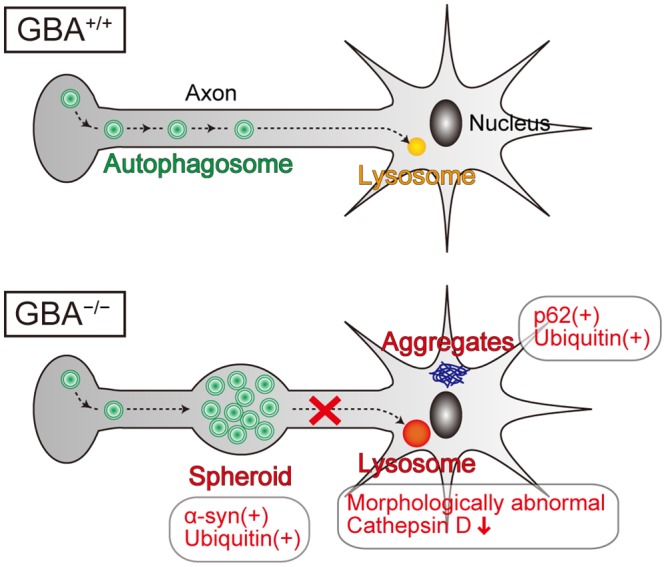
Pathological findings and proposed pathological mechanisms in neurons of *GBA* ^-/-^ medaka. Neurons of *GBA* ^-/-^ medaka showed lysosomal dysfunction, which is reflected in morphologically abnormal structures with decreased Cathepsin D staining intensity, and axonal swellings containing autophagosomes where α-syn and ubiquitin accumulate. p62-positive aggregates, which colocalize with ubiquitin, are not located in axonal swellings, but presumably in soma or dendrites. Considering the previous studies about the axonal transport of autophagosomes, we propose that GCase deficiency primarily causes lysosomal dysfunction, which leads to disrupted retrograde transport of autophagosomes in axons and formation of axonal swellings with α-syn accumulation.

Lysosomes play a fundamental role in the autophagic pathway by fusing with autophagosomes and digesting their contents[45]. In general, lysosomal dysfunction results in defective digestion and accumulation of autophagic substrates such as polyubiquitinated proteins, p62, and dysfunctional mitochondria, accompanied by accumulation of autophagosomes. Axonal swellings containing autophagosome-like structures are observed in mouse models of lysosomal storage diseases such as neuronopathic GD mice, Niemann-pick type C1 mice, Cathepsin D-deficient mice, and Cathepsin B/L double-deficient mice[34,46,47]. A previous study reported that autophagosomes are formed in the distal part of the axon and undergo retrograde transport toward the cell body[48]. Another previous study demonstrated that inhibition of lysosomal function in primary neurons from mouse embryos disrupts the axonal transport of autophagosomes and causes their accumulation in axons[49]. Considering these lines of evidence, we propose that GCase deficiency primarily causes lysosomal dysfunction, leading to disrupted retrograde transport of autophagosomes in axons and formation of axonal swellings with α-syn accumulation (Fig. 6). Contrary to our expectations, p62-positive aggregates did not colocalize with LC3- and α-syn-positive axonal swellings, but were mainly located in the perikarya of neurons. These results suggest the possibility that presynaptic α-syn is transported proximally and degraded in a p62-independent autophagy-lysosome pathway. A previous study supports this hypothesis. Conditional knockout mice lacking ATG7 in TH-positive neurons show α-syn aggregates in swollen axons in the striatum, but not in cell bodies in the midbrain, indicating that autophagic dysfunction initially causes α-syn aggregates in the distal part of axons[50]. In addition, another previous study demonstrated that α-syn aggregation occurs earlier in axons than in neuronal cell bodies in the cardiac sympathetic nervous system in PD patients[51]. Because axonal retrograde transport may be involved in the degradative pathway of α-syn and may be a therapeutic target in PD, further studies are required to elucidate the precise mechanisms.

Because *GBA* ^+/-^ medaka as old as 12 months did not show any apparent abnormal phenotypes, we could not directly investigate how heterozygous *GBA* mutations cause PD. Meanwhile, according to a recent report, induced pluripotent stem cell-derived neurons from PD patients carrying heterozygous *GBA* mutations show α-syn accumulation, an impaired autophagy-lysosome pathway, and dysregulation of calcium homeostasis[12]. The reason for differences in phenotypes between *in vivo* and *in vitro* models is unclear. However, our findings from *GBA* ^+/-^ medaka seem to be reasonable because the penetrance of PD in *GBA* mutation carriers is estimated to be at most 30% by the age of 80 years[52]. Thus, second hits such as environmental factors and other genetic factors are probably required for the development of PD pathology *in vivo*.

Cellular and animal PD models overexpressing α-syn have provided evidence for the various potential toxic mechanisms of α-syn. However, few studies have demonstrated the pathological role of endogenous α-syn *in vivo*, which may reflect the authentic role for α-syn in PD. A previous study showed that *α-syn* null mice are resistant to 1-methyl-4-phenyl-1,2,3,6-tetrahydropyridine (MPTP) toxicity compared with wild-type mice[53]. However, it is unclear whether this improvement is due to attenuation of α-syn toxicity or α-syn-mediated changes in the presynaptic machinery. Another previous study showed that the formation of intraneuronal inclusions and neurodegeneration in 26S proteasome-depleted mice is independent of α-syn[54]. In the present study, disruption of *α-syn* did not improve the life span, neuronal loss, or neuroinflammation in *GBA* ^-/-^ medaka. Moreover, α-syn was not involved in the accumulation of autophagosomes in axons. Our data indicate that α-syn accumulation is a downstream event, and other severe pathological factors may obscure the involvement of α-syn in the pathogenesis of neuronopathic GD in medaka.

In summary, the present study showed that *GBA* ^-/-^ medaka are useful as a viable neuronopathic GD model with endogenous α-syn accumulation. Long-term survival of these fish allows us to observe the pathological progression. Our data revealed that GCase deficiency causes lysosomal dysfunction in neurons and α-syn accumulation in axonal swellings containing autophagosomes. Axonal transport of α-syn may play an important role in the mechanisms of *GBA* mutations leading to PD and may also be a therapeutic target in PD. Furthermore, we demonstrate the minimal contribution of α-syn to the pathogenesis of neuronopathic GD in medaka. *GBA* ^-/-^ medaka represent a valuable model for exploring the pathological mechanisms and also provide a new platform for developing treatments in PD with *GBA* mutations as well as neuronopathic GD.

## Materials and Methods

### Ethics statement

Medaka were anesthetized in 0.02% tricaine in fish water and then sacrificed. All experimental procedures used in this study followed national guidelines. The Animal Research Committee of Kyoto University granted a formal waiver of ethical approval and also granted permission.

### Maintenance of medaka

Medaka of the Kyoto-cab strain, a substrain of Cab, were maintained at 27°C in a recirculating aquaculture system. Adult fish were kept in a reproduction regimen (14 hr light/10 hr dark). Eggs were kept in a dark box at 28°C.

### Cloning of medaka genes

RNA was extracted from wild-type medaka brains with Qiazol (QIAGEN) according to the manufacturer’s instructions. cDNA was synthesized using the PrimeScript RT reagent kit (Perfect Real Time) (TaKaRa, #RR037A). To identify medaka *GBA*, *SNCA*, *SNCGb*, *p62/SQSTM1*, and *MAP1LC3B* orthologs, we referred to the medaka genome database (http://www.ensembl.org/Oryzias_latipes/Info/Index). Because their cDNA sequences and amino acid sequences were not completely known, we determined their cDNA sequences using a combination of RT-PCR and rapid amplification of cDNA ends, the products of which were generated using Seegene’s Capfishing kit (Seegene). The cDNA sequences can be found in the European Nucleotide Archive (ENA, accession numbers LM644999–645003).

### Generation of *GBA* nonsense mutant medaka

*GBA* mutant medaka were generated as described[17]. To find *GBA* mutations in the TILLING library, we narrowed down mutated candidates from 5,771 samples using a high-resolution melting assay, followed by determination of the DNA sequences[24]. We screened the TILLING library for exons 1–2, exons 5–7, exon 8, and exons 9–11 of *GBA*. *In vitro* fertilization was carried out using sperm from a sample with the favorable mutation. To genotype the progeny of *GBA* ^*WT/W337X*^ mutants, PCR was performed with the following primer set (5′-AGGGTTGAAGGGGTTAAGCA-3′, 5′-TTGTAACCAGTACCGCAGCA-3′), designed HybProbes (5′-LC Red 640-CATGTACCAGTGGACG-Phosphate-3′, 5′-CCTAAGCTTATATCTGCAGGGACTAAACTGT-Fluorescein-3′), and LightCycler 480 Probes Master in LightCycler 480 (Roche) according to the manufacturer's instructions. The genotypes can be distinguished with high-resolution melting curve analysis. *GBA* ^*WT/W337X*^ mutants were back-crossed to Kyoto-Cab medaka at least seven times and then crossed to obtain *GBA* ^*W337X/W337X*^ mutants.

### Rescue experiment

For the rescue experiment, we established *GBA* transgenic medaka in which medaka *GBA* expression was driven by the medaka *GAP-43* promoter (*Tg(GAP-43*:*GBA*)). We used an insulator located in the upstream region of sea urchin (*Hemicentrotus pulcherrimus*) *arylsulfatase*[55,56]. The transgenic construct was flanked by two insulators and included the medaka *GAP-43* promoter followed by an internal ribosome entry site (IRES), enhanced GFP (EGFP), and the Simian virus 40 polyadenylation site or the medaka *β-actin* 3′-untranslated region (3′UTR) (S8A Fig). The medaka *GAP-43* promoter contained a 1.0-kb fragment of the 5′-flanking region of the gene. A DNA fragment of the transgene was inserted into EcoR I/Sal I restriction enzyme sites of the pDs-actb2k-EGFP plasmid. pDs-actb2k-EGFP was constructed by inserting an Xho I/Spe I fragment from pactb2k-EGFP into the Xho I/Spe I site of the pDs-GTDEL4 plasmid, which contains 5′- and 3′-Ds sequences[21,57]. The resultant vector was injected into the cytoplasm of fertilized Kyoto-Cab eggs before the first cleavage as described[20].

### Generation of *α-syn* deletion mutant medaka

*α-syn* deletion mutant medaka were generated with TALENs as described[58]. To genotype the progeny of *α-syn* deletion mutants, PCR was performed with the following primer set (5′-GATCCCGAGCCATCCAC-3′, 5′-TGCAACTGTGGAAACACCAT-3′), followed by electrophoresis in a 10% (w/v) polyacrylamide gel (S4D Fig). RT-PCR for *α-syn* was performed with the following primer sets (*α-syn*: 5′-GATCCCGAGCCATCCAC-3′, 5′-TTTGGAGAAACCCTTCATTAAC-3′; *β-actin*: 5′-TCCACCTTCCAGCAGATGTG-3′, 5′-AGCATTTGCGGTGGACGAT-3′) (S4E Fig).

### Behavioral analyses

The spontaneous swimming movement of medaka was traced using a video camera positioned above the water tank and analyzed with ethovision XT 5 (Noldus). The water tank was a transparent circular container (20 cm diameter, 2 cm water depth, room temperature). Image acquisition began 5 min after medaka were placed in a new water tank. Data were collected continuously for the subsequent 3 min.

### GCase enzymatic activity assay

The assay for GCase enzymatic activity was performed as described[11]. Medaka brains were homogenized in 40 μl sample buffer (10 mM Tris-HCl, 150 mM NaCl, 1% (v/v) Triton X-100, pH 7.4), sonicated, and centrifuged at 10,000 ×*g* at 4°C for 5 min. Aliquots containing 50 μg protein were incubated in assay buffer (5 mM 4-Methylumbelliferyl β-d-glucopyranoside (Wako, #324–37441), 1% (w/v) sodium taurocholate (Wako, #197–10033), 50 mM sodium citrate, 50 mM sodium phosphate, pH 5.0) in the presence or absence of 2 mM Conduritol B epoxide (Toronto Research Chemicals, #C666000) in a total volume of 100 μl at 37°C for 4 hr. The reaction was stopped by adding 100 μl of 0.4 M glycine, pH 10.8, and the fluorescence at 460 nm (emission 355 nm) was measured with Fluoroskan Ascent FL (Thermo Fisher).

### Lipid analyses with supercritical fluid chromatography (SFC)/mass spectrometry (MS)/MS

Medaka brains were stored at—80°C until analyses and homogenized in 1 ml tissue homogenization buffer (250 mM sucrose, 25 mM KCl, 50 mM Tris-HCl, 0.5 mM EDTA, pH 7.4). Aliquots containing 1 mg protein were used for the analyses. Levels of glucocerebroside and galactocerebroside were measured with high-performance liquid chromatography-tandem MS as described with modification using SFC separation[59].

### Generation of the medaka α-syn antibody

A synthetic peptide corresponding to amino acids 90–104 of medaka α-syn conjugated to Keyhole Limpet Hemocyanin was used for immunization of rabbits. Serum was obtained 49 days after immunization and purified with affinity chromatography.

### Immunohistochemical analyses

Frozen and paraffin sections were used for immunohistochemical analyses. For frozen sections, medaka brains were fixed with 4% (w/v) paraformaldehyde (PFA) in phosphate-buffered saline (PBS) at 4°C for 4 hr and then placed in 30% (w/v) sucrose at 4°C for more than 16 hr. Samples were embedded in Surgipath FSC 22 (Leica), and 14-μm-thick sections were obtained with a LEICA CM 1900. For paraffin sections, medaka brains were fixed in 4% (w/v) PFA at 4°C for 16 hr, dehydrated, and embedded in paraffin. Sections with a thickness of 6 to 20 μm were obtained with a Microm HM 325. For immunohistochemical analyses, the following primary antibodies and a lectin were used: anti-Cathepsin D (Calbiochem, #IM03, 1:200), anti-GFAP (Sigma-Aldrich, #G3893, 1:1000), anti-LC3 (Santa Cruz, #sc-16755, 1:100), anti-medaka α-syn (1:2000), anti-p62 (MBL, #PM045, 1:2000), anti-ssDNA (DAKO, #A4506, 1:2000), anti-TH (Millipore, #MAB318, 1:1000), anti-TPH (Abcam, #ab3907–50, 1:1000), anti-ubiquitin (Santa Cruz, #sc-8017, 1:50), anti-ubiquitin (DAKO, #Z0458, 1:1000), and biotinylated Lycopersicon Esculentum (Tomato) Lectin (VECTOR, #B-1175, 5 <g/ml). The sections were incubated at 4°C with primary antibodies or lectin for 1 to 3 days and then processed for visualization. As secondary antibodies, ImmPRESS (VECTOR) was used for diaminobenzidine staining, and Alexa Fluor-conjugated antibodies (Molecular Probes) were used for immunofluorescence. Sections were observed with an Olympus CX41 microscope, a BZ-9000 fluorescence microscope (KEYENCE), and an Olympus FV-1000 confocal laser scanning microscope. For confocal microscope images, Pearson's coefficient correlation (*r*) was calculated using Olympus software.

### Measurement of mRNA in medaka brains

RNA was extracted from medaka brains with Qiazol (QIAGEN) according to the manufacturer’s instructions. cDNA was generated with reverse transcription using the PrimeScript RT reagent kit (Perfect Real Time) (TaKaRa, #RR037A). The amount of cDNA was quantified with real-time PCR using LightCycler 480 SYBR Green I Master (Roche, #04887352001) and Roche LightCycler 480. The following primer sets were used (*TNFα*: 5′-ATTGGAGTGAAAGGCCAGAA-3′, 5′-ACTAATTTGAGACCGCCACG-3′; *β-actin*: 5′-TCCACCTTCCAGCAGATGTG-3′, 5′-AGCATTTGCGGTGGACGAT-3′).

### In situ hybridization

The vector including a portion of the medaka *APOE* cDNA sequence was generously provided by Dr. H. Mitani (Tokyo University, Tokyo, Japan) (in submission). In situ hybridization was performed as described[60]. Counterstaining was performed with methyl green (Sigma-Aldrich, #M8884).

### Western blot analyses

For Triton-soluble fractions, samples were homogenized in high-salt buffer containing 1% Triton X-100 (750 mM NaCl, 5 mM EDTA, 50 mM Tris-HCl, 1% (v/v) Triton X-100, pH 7.5) and centrifuged at 20,400 ×*g* at 4°C for 5 min. For SDS-soluble fractions, the pellet was subsequently sonicated in SDS buffer (50 mM Tris-HCl, 2% SDS, pH 7.4) followed by centrifugation at 20,400 ×*g* at 4°C for 5 min. The supernatant was boiled in sample buffer (1% (w/v) SDS, 12.5% (w/v) glycerol, 0.005% (w/v) bromophenol blue, 2.5% (v/v) 2-mercaptoethanol, 25 mM Tris-HCl, pH 6.8). Samples were separated on 10%, 12%, or 14% (w/v) gels for SDS-PAGE. Samples containing 20 μg protein were loaded in each lane for Triton-soluble fractions, and SDS-soluble fractions were loaded according to the amount of protein in Triton-soluble fractions. Proteins were transferred to polyvinylidene difluoride membranes with a Trans-Blot SD Semi-Dry Transfer Cell (Bio-Rad). For detection of medaka α-syn and mouse α-syn, the membranes were treated with 0.4% (w/v) PFA in PBS for 30 min at room temperature before blocking to prevent detachment of α-syn from the blotted membranes[61]. For western blot analyses, the following primary antibodies were used: anti-β-actin (Sigma-Aldrich, #A1978, 1:5000), anti-GFAP (Sigma-Aldrich, #G3893, 1:1000), anti-LC3 (MBL, #PM036, 1:2000), anti-NSE (DAKO, #M0873, 1:500), anti-α-syn (BD Transduction, #610787, 1:2000), anti-medaka α-syn (1:10,000), anti-phosphorylated neurofilament (COVANCE, #smi-31r, 1:1000), anti-p62 (MBL, #PM045, 1:500), anti-TH (Millipore, #MAB318, 1:1000), and anti-ubiquitin (Santa Cruz, #sc-8017, 1:50). The membranes were incubated with primary antibodies for 1 to 3 days at 4°C, followed by reaction with horseradish peroxidase-conjugated secondary antibodies (Santa Cruz) for 1 hr at room temperature. Immunoreactive bands were detected with Chemi-Lumi One Super (Nacalai tesque), and the chemiluminescent signal was detected with Fujifilm LAS-3000. Densitometric analyses were performed using ImageJ software (National Institutes of Health).

### High performance liquid chromatography

To measure the amounts of dopamine, noradrenaline and serotonin in medaka brains, high performance liquid chromatography was performed as described[60].

### Transmission electron microscopy and toluidine blue staining

Medaka brains were fixed with 4% (w/v) PFA and 2% (v/v) glutaraldehyde (Wako, #072–01961) in 0.1 M phosphate buffer (PB) at 4°C for 16 hr. After rinsing in 0.1 M PB, samples were postfixed with 1% (w/v) OsO_4_ in 0.1 M PB for 2 hr. Then, samples were dehydrated, penetrated with ethanol and a propylene oxide series, and embedded in Epon. Sections were obtained with an EM UC6 ultramicrotome (Leica). Sections with a thickness of 1 μm were used for toluidine blue staining. Sections with a thickness of 60 to 80 nm were stained with uranyl acetate and lead citrate and observed with a Hitachi H-7650 transmission electron microscope.

### Immunoelectron microscopy

Immunoelectron microscopy using ultrathin cryosections was performed as described[62]. Briefly, brains were quickly removed from medaka and immersed in 4% PFA buffered with 0.1 M PB (pH 7.2) at 4°C for 2 hr, washed thoroughly with 7.5% sucrose in 0.1 M PB (pH 7.2), and embedded in 12% gelatin. The samples were rotated in 2.3 M sucrose in 0.1 M PB overnight at 4°C, placed on a specimen holder (Leica Microsystems), and quickly plunged into liquid nitrogen. Ultrathin cryosections were cut with a Leica UC6/FC6 and UC7/FC7 (Leica Microsystems) at about—120°C. Sections about 60 nm thick were picked up with a 1:1 mixture of 2% methylcellulose and 2.3 M sucrose and transferred to a nickel grid with a carbon-coated Formvar supporting film. The sections were rinsed with PBS containing 0.02 M glycine, treated with 1% bovine serum albumin in PBS, and incubated overnight at 4°C with rabbit anti-medaka α-syn antibody (1:30). They were then incubated with goat anti-rabbit IgG conjugated to 10 nm colloidal gold particles (1:40) (British Biocell International) for 1 hr at room temperature. Immunostained sections were fixed with 1% glutaraldehyde in PBS. After completion of the labeling, the sections were embedded in a thin layer of 2% methylcellulose with 0.4% uranyl acetate (pH 4.0), air-dried, and observed with a Hitachi H-7100 electron microscope. For control experiments, ultrathin sections were reacted only with the gold particle-conjugated secondary antibody.

### Statistical analyses

A two-tailed paired Student’s t-test or one-way ANOVA with Tukey’s post-hoc test was used for analyses. Statistical calculations were performed with Microsoft Excel or GraphPad Prism Software, Version 5.0.

## Supporting Information

S1 Fig Sequence alignment of GBA. Sequence alignment of human (*Homo sapiens*), mouse (*Mus musculus*), and medaka (*Oryzias latipes*) GBA protein. Amino acids conserved in two or three species are outlined. The red outlining indicates the W337X mutation in the *GBA* nonsense mutant medaka.
(TIF)

S2 Fig N-acyl chain distribution of glucocerebroside and galactocerebroside. N-acyl chain distribution of glucocerebroside and galactocerebroside in medaka brains at 3 months (n = 3–4). For all analyses, data are the mean ± SEM.
(TIF)

S3 Fig Periodic acid-Schiff (PAS)-positive abnormal cells in the spleen and kidney of *GBA* ^-/-^ medaka. PAS staining of medaka spleen and kidney at 3 months. Clusters of abnormal PAS-positive cells in the spleen and kidney (arrowheads) and high-magnification images (right panels). Scale bars of right panels, 20 μm. Other scale bars, 50 μm.
(TIF)

S4 Fig Specificity of medaka α-syn antibody. (A) Sequence alignment of human and medaka α-syn protein. Conserved amino acids are outlined. The red outlining indicates the epitope of medaka α-syn antibody. (B) Sequence alignment of medaka α-synuclein (SNCA), β-synuclein (SNCB), γ-synuclein-a (SNCGa), and γ-synuclein-b (SNCGb). Amino acids conserved in three or four proteins are outlined. The red outlining indicates the epitope of the medaka α-syn antibody. (C) Upper panel: DNA sequences of wild-type *α-syn* and deleted *α-syn*. Lower panel: Sequence alignment of wild-type α-syn and mutated α-syn. The red outlining indicates the epitope of the medaka α-syn antibody. (D) Polyacrylamide gel electrophoresis (PAGE) image of PCR for *α-syn*. PCR primers were designed to span the deleted region of *α-syn*, allowing the genotypes to be distinguished with PAGE. (E) RT-PCR for *α-syn* mRNA. One primer was designed to overlap the deleted region of *α-syn*. Intact *α-syn* mRNA was not detected in *α-syn* ^-/-^ medaka. (F) Western blot analysis of α-syn and β-actin. A 14-kDa putative α-syn band was observed only in *α-syn* ^*+/+*^ medaka, suggesting the authenticity of the medaka α-syn antibody. (G) Immunohistochemistry with medaka α-syn antibody. α-syn immunostaining was observed only in *α-syn* ^*+/+*^ medaka. Scale bars, 50 μm. (H) Immunoelectron micrograph of a presynaptic region with immunogold-labeled α-syn. Left panels: presynaptic area with small synaptic vesicles. Right panels: presynaptic area with large synaptic vesicles. The postsynaptic density is visible (arrowheads). Scale bars, 200 nm.
(TIF)

S5 Fig α-syn accumulation was observed in *GBA* ^-/-^ medaka at 2 months, not at 1 month. α-syn immunohistochemistry at 1 and 2 months after fertilization. α-syn accumulation was observed in *GBA* ^-/-^ medaka at 2 months (arrowheads). Scale bars, 20 μm.
(TIF)

S6 Fig Transmission electron micrographs of neuropil and co-localization analysis of axonal swellings in *GBA* ^-/-^ medaka. (A) Transmission electron micrographs of neuropil. Left and middle panels: Neuropil of a *GBA* ^*+/+*^ and a *GBA* ^-/-^ medaka, respectively. Swellings of both myelinated (arrowheads) and unmyelinated (outlined by dashed lines) axons were found in *GBA* ^-/-^ medaka, which contain vacuoles and electron-dense bodies. Scale bars, 2 μm. Right panels: High-magnification images of swellings of myelinated and unmyelinated axons (lower and upper panels, respectively). Scale bars, 500 nm. (B) Co-localization analysis for different markers in axonal swellings of *GBA* ^-/-^ medaka. The correlation between LC3, ubiquitin, and α-syn signals are shown as the pixel scatter diagrams and a graph (n = 6). For all analyses, data are the mean ± SEM.
(TIF)

S7 Fig Analyses of *GBA* ^+/−^ medaka at 12 months. (A) α-syn immunohistochemistry at 12 months. α-syn accumulation was not observed in *GBA* ^+/-^ medaka. Scale bars, 20 μm. (B) Western blot analysis of α-syn and β-actin (n = 5–6). (C) Numbers of TH-positive neurons in the middle diencephalon and TH-positive neurons in the locus coeruleus at 12 months (n = 4). (D) Total swimming distance at 12 months (n = 12). (E) Amounts of dopamine, noradrenaline, and serotonin in the brains at 12 months measured with high performance liquid chromatography. All values are expressed as the amount (μg) per protein (mg) (n = 12). For all analyses, data are the mean ± SEM. A two-tailed paired Student’s t-test was used to determine the statistical significance.
(TIF)

S8 Fig Localization and co-localization analyses in the brains of *GBA* ^-/-^ medaka. (A) Double immunostaining in *GBA* ^-/-^ medaka at 3 months. Upper panels: NeuN (green), GFAP (green), or LEL (green) and p62 (red). Lower panels: NeuN (green), GFAP (green), or LEL (green) and ubiquitin (red). Nuclei were visualized with DAPI (blue). p62- and Ubiquitin-positive aggregates were localized only in NeuN-positive neurons, but not in GFAP-positive radial glial cells or LEL-positive microglia. Scale bars, 20 μm. (B) Co-localization analysis for different markers in the brains of *GBA* ^-/-^ medaka. The correlation between LC3, ubiquitin, and α-syn signals are shown as the pixel scatter diagrams and a graph (n = 6). For all analyses, data are the mean ± SEM.
(TIF)

S9 Fig Transgenic expression of *GBA* reversed the pathological phenotypes of *GBA* ^-/-^ medaka. (A) Transgenic construct used to establish *GBA* transgenic medaka. (B) GCase activity in the brains of each *Tg(GAP-43*:*GBA);GBA* ^-/-^ lines (described as Tg-line No.;*GBA* ^-/-^ in the figure) at 3 months (n = 5–6). (C) Total swimming distance during 3 min in *GBA* ^*+/+*^, *GBA* ^-/-^, and *Tg(GAP-43*:*GBA)line3;GBA* ^-/-^ medaka (n = 8, *p < 0.05). (D) Hematoxylin and eosin staining, *APOE* in situ hybridization, and p62 and α-syn immunohistochemistry of *Tg(GAP-43*:*GBA)line3;GBA* ^-/-^ medaka showed no major abnormalities. Scale bars, 100 μm, 50 μm, 10 μm, 20 μm, respectively. For all analyses, data are the mean ± SEM.
(TIF)

S1 Movie Swimming movement of *GBA* ^+/+^ medaka at 2 months. (MP4)

S2 Movie Swimming movement of *GBA* ^-/-^ medaka at 2 months. (MP4)

S3 Movie Swimming movement of *Tg(GAP-43*:*GBA)line3;GBA* ^-/-^ medaka at 2 months. (MP4)

## References

[1] Futerman AH, Zimran A (2006) Gaucher disease: CRC Press.

[2] Grabowski GA (2008) Phenotype, diagnosis, and treatment of Gaucher's disease. Lancet 372: 1263–1271. 10.1016/S0140-6736(08)61522-6 19094956

[3] Wong K, Sidransky E, Verma A, Mixon T, Sandberg GD, et al (2004) Neuropathology provides clues to the pathophysiology of Gaucher disease. Mol Genet Metab 82: 192–207. 15234332 10.1016/j.ymgme.2004.04.011

[4] Eblan MJ, Goker-Alpan O, Sidransky E (2005) Perinatal lethal Gaucher disease: a distinct phenotype along the neuronopathic continuum. Fetal Pediatr Pathol 24: 205–222. 16396828 10.1080/15227950500405296

[5] Neumann J, Bras J, Deas E, O'Sullivan SS, Parkkinen L, et al (2009) Glucocerebrosidase mutations in clinical and pathologically proven Parkinson's disease. Brain 132: 1783–1794. 10.1093/brain/awp044 19286695 PMC2702833

[6] Sidransky E, Nalls MA, Aasly JO, Aharon-Peretz J, Annesi G, et al (2009) Multicenter analysis of glucocerebrosidase mutations in Parkinson's disease. N Engl J Med 361: 1651–1661. 10.1056/NEJMoa0901281 19846850 PMC2856322

[7] Bultron G, Kacena K, Pearson D, Boxer M, Yang R, et al (2010) The risk of Parkinson's disease in type 1 Gaucher disease. J Inherit Metab Dis 33: 167–173. 10.1007/s10545-010-9055-0 20177787 PMC2887303

[8] Manning-Bog AB, Schule B, Langston JW (2009) Alpha-synuclein-glucocerebrosidase interactions in pharmacological Gaucher models: a biological link between Gaucher disease and parkinsonism. Neurotoxicology 30: 1127–1132. 10.1016/j.neuro.2009.06.009 19576930

[9] Mazzulli JR, Xu YH, Sun Y, Knight AL, McLean PJ, et al (2011) Gaucher disease glucocerebrosidase and alpha-synuclein form a bidirectional pathogenic loop in synucleinopathies. Cell 146: 37–52. 10.1016/j.cell.2011.06.001 21700325 PMC3132082

[10] Sardi SP, Clarke J, Kinnecom C, Tamsett TJ, Li L, et al (2011) CNS expression of glucocerebrosidase corrects alpha-synuclein pathology and memory in a mouse model of Gaucher-related synucleinopathy. Proc Natl Acad Sci U S A 108: 12101–12106. 10.1073/pnas.1108197108 21730160 PMC3141921

[11] Xu YH, Sun Y, Ran H, Quinn B, Witte D, et al (2011) Accumulation and distribution of alpha-synuclein and ubiquitin in the CNS of Gaucher disease mouse models. Mol Genet Metab 102: 436–447. 10.1016/j.ymgme.2010.12.014 21257328 PMC3059359

[12] Schondorf DC, Aureli M, McAllister FE, Hindley CJ, Mayer F, et al (2014) iPSC-derived neurons from GBA1-associated Parkinson's disease patients show autophagic defects and impaired calcium homeostasis. Nat Commun 5: 4028 10.1038/ncomms5028 24905578

[13] Gegg ME, Burke D, Heales SJ, Cooper JM, Hardy J, et al (2012) Glucocerebrosidase deficiency in substantia nigra of parkinson disease brains. Ann Neurol 72: 455–463. 10.1002/ana.23614 23034917 PMC3638323

[14] Sardi SP, Singh P, Cheng SH, Shihabuddin LS, Schlossmacher MG (2012) Mutant GBA1 expression and synucleinopathy risk: first insights from cellular and mouse models. Neurodegener Dis 10: 195–202. 10.1159/000335038 22327140

[15] Murphy KE, Gysbers AM, Abbott SK, Tayebi N, Kim WS, et al (2014) Reduced glucocerebrosidase is associated with increased alpha-synuclein in sporadic Parkinson's disease. Brain 137: 834–848. 10.1093/brain/awt367 24477431 PMC3927701

[16] Wittbrodt J, Shima A, Schartl M (2002) Medaka—a model organism from the far East. Nat Rev Genet 3: 53–64. 11823791 10.1038/nrg704

[17] Taniguchi Y, Takeda S, Furutani-Seiki M, Kamei Y, Todo T, et al (2006) Generation of medaka gene knockout models by target-selected mutagenesis. Genome Biol 7: R116 17156454 10.1186/gb-2006-7-12-r116 PMC1794429

[18] Ansai S, Kinoshita M (2014) Targeted mutagenesis using CRISPR/Cas system in medaka. Biol Open 3: 362–371. 10.1242/bio.20148177 24728957 PMC4021358

[19] Ansai S, Sakuma T, Yamamoto T, Ariga H, Uemura N, et al (2013) Efficient targeted mutagenesis in medaka using custom-designed transcription activator-like effector nucleases. Genetics 193: 739–749. 10.1534/genetics.112.147645 23288935 PMC3583995

[20] Kinoshita M, Kani S, Ozato K, Wakamatsu Y (2000) Activity of the medaka translation elongation factor 1alpha-A promoter examined using the GFP gene as a reporter. Dev Growth Differ 42: 469–478. 11041488 10.1046/j.1440-169x.2000.00530.x

[21] Ansai S, Ochiai H, Kanie Y, Kamei Y, Gou Y, et al (2012) Targeted disruption of exogenous EGFP gene in medaka using zinc-finger nucleases. Dev Growth Differ 54: 546–556. 10.1111/j.1440-169X.2012.01357.x 22642582

[22] Matsui H, Gavinio R, Asano T, Uemura N, Ito H, et al (2013) PINK1 and Parkin complementarily protect dopaminergic neurons in vertebrates. Hum Mol Genet 22: 2423–2434. 10.1093/hmg/ddt095 23449626 PMC10259650

[23] Matsui H, Sato F, Sato S, Koike M, Taruno Y, et al (2013) ATP13A2 deficiency induces a decrease in cathepsin D activity, fingerprint-like inclusion body formation, and selective degeneration of dopaminergic neurons. FEBS Lett 587: 1316–1325. 10.1016/j.febslet.2013.02.046 23499937

[24] Ishikawa T, Kamei Y, Otozai S, Kim J, Sato A, et al (2010) High-resolution melting curve analysis for rapid detection of mutations in a Medaka TILLING library. BMC Mol Biol 11: 70 10.1186/1471-2199-11-70 20840787 PMC2949603

[25] Tayebi N, Cushner SR, Kleijer W, Lau EK, Damschroder-Williams PJ, et al (1997) Prenatal lethality of a homozygous null mutation in the human glucocerebrosidase gene. Am J Med Genet 73: 41–47. 9375921 10.1002/(sici)1096-8628(19971128)73:1<41::aid-ajmg9>3.0.co;2-s

[26] Tybulewicz VL, Tremblay ML, LaMarca ME, Willemsen R, Stubblefield BK, et al (1992) Animal model of Gaucher's disease from targeted disruption of the mouse glucocerebrosidase gene. Nature 357: 407–410. 1594045 10.1038/357407a0

[27] Enquist IB, Lo Bianco C, Ooka A, Nilsson E, Mansson JE, et al (2007) Murine models of acute neuronopathic Gaucher disease. Proc Natl Acad Sci U S A 104: 17483–17488. 17954912 10.1073/pnas.0708086104 PMC2077282

[28] Pennelli N, Scaravilli F, Zacchello F (1969) The morphogenesis of Gaucher cells investigated by electron microscopy. Blood 34: 331–347. 5804023

[29] Sun Y, Quinn B, Witte DP, Grabowski GA (2005) Gaucher disease mouse models: point mutations at the acid beta-glucosidase locus combined with low-level prosaposin expression lead to disease variants. J Lipid Res 46: 2102–2113. 16061944 10.1194/jlr.M500202-JLR200

[30] Peri F, Nusslein-Volhard C (2008) Live imaging of neuronal degradation by microglia reveals a role for v0-ATPase a1 in phagosomal fusion in vivo. Cell 133: 916–927. 10.1016/j.cell.2008.04.037 18510934

[31] Baumgart EV, Barbosa JS, Bally-Cuif L, Gotz M, Ninkovic J (2012) Stab wound injury of the zebrafish telencephalon: a model for comparative analysis of reactive gliosis. Glia 60: 343–357. 10.1002/glia.22269 22105794

[32] Farfel-Becker T, Vitner EB, Pressey SN, Eilam R, Cooper JD, et al (2011) Spatial and temporal correlation between neuron loss and neuroinflammation in a mouse model of neuronopathic Gaucher disease. Hum Mol Genet 20: 1375–1386. 10.1093/hmg/ddr019 21252206

[33] Rink E, Wullimann MF (2001) The teleostean (zebrafish) dopaminergic system ascending to the subpallium (striatum) is located in the basal diencephalon (posterior tuberculum). Brain Res 889: 316–330. 11166725 10.1016/s0006-8993(00)03174-7

[34] Sun Y, Liou B, Ran H, Skelton MR, Williams MT, et al (2010) Neuronopathic Gaucher disease in the mouse: viable combined selective saposin C deficiency and mutant glucocerebrosidase (V394L) mice with glucosylsphingosine and glucosylceramide accumulation and progressive neurological deficits. Hum Mol Genet 19: 1088–1097. 10.1093/hmg/ddp580 20047948 PMC2830832

[35] Vitner EB, Dekel H, Zigdon H, Shachar T, Farfel-Becker T, et al (2010) Altered expression and distribution of cathepsins in neuronopathic forms of Gaucher disease and in other sphingolipidoses. Hum Mol Genet 19: 3583–3590. 10.1093/hmg/ddq273 20616152

[36] Cuoghi B, Mola L (2007) Microglia of teleosts: facing a challenge in neurobiology. Eur J Histochem 51: 231–240. 18162452

[37] Fujimori KE, Kawasaki T, Deguchi T, Yuba S (2008) Characterization of a nervous system-specific promoter for growth-associated protein 43 gene in Medaka (Oryzias latipes). Brain Res 1245: 1–15. 10.1016/j.brainres.2008.09.071 18951884

[38] Dawson TM, Ko HS, Dawson VL (2010) Genetic animal models of Parkinson's disease. Neuron 66: 646–661. 10.1016/j.neuron.2010.04.034 20547124 PMC2917798

[39] Hirsch EC, Hunot S (2009) Neuroinflammation in Parkinson's disease: a target for neuroprotection? Lancet Neurol 8: 382–397. 10.1016/S1474-4422(09)70062-6 19296921

[40] Kim C, Ho DH, Suk JE, You S, Michael S, et al (2013) Neuron-released oligomeric alpha-synuclein is an endogenous agonist of TLR2 for paracrine activation of microglia. Nat Commun 4: 1562 10.1038/ncomms2534 23463005 PMC4089961

[41] Abeliovich A, Schmitz Y, Farinas I, Choi-Lundberg D, Ho WH, et al (2000) Mice lacking alpha-synuclein display functional deficits in the nigrostriatal dopamine system. Neuron 25: 239–252. 10707987 10.1016/s0896-6273(00)80886-7

[42] Farfel-Becker T, Vitner EB, Futerman AH (2011) Animal models for Gaucher disease research. Dis Model Mech 4: 746–752. 10.1242/dmm.008185 21954067 PMC3209644

[43] Holleran WM, Ginns EI, Menon GK, Grundmann JU, Fartasch M, et al (1994) Consequences of beta-glucocerebrosidase deficiency in epidermis. Ultrastructure and permeability barrier alterations in Gaucher disease. J Clin Invest 93: 1756–1764. 8163674 10.1172/JCI117160 PMC294236

[44] Murayama E, Kissa K, Zapata A, Mordelet E, Briolat V, et al (2006) Tracing hematopoietic precursor migration to successive hematopoietic organs during zebrafish development. Immunity 25: 963–975. 17157041 10.1016/j.immuni.2006.10.015

[45] Lieberman AP, Puertollano R, Raben N, Slaugenhaupt S, Walkley SU, et al (2012) Autophagy in lysosomal storage disorders. Autophagy 8: 719–730. 10.4161/auto.19469 22647656 PMC3378416

[46] Liao G, Yao Y, Liu J, Yu Z, Cheung S, et al (2007) Cholesterol accumulation is associated with lysosomal dysfunction and autophagic stress in Npc1 -/- mouse brain. Am J Pathol 171: 962–975. 17631520 10.2353/ajpath.2007.070052 PMC1959498

[47] Koike M, Shibata M, Waguri S, Yoshimura K, Tanida I, et al (2005) Participation of autophagy in storage of lysosomes in neurons from mouse models of neuronal ceroid-lipofuscinoses (Batten disease). Am J Pathol 167: 1713–1728. 16314482 10.1016/S0002-9440(10)61253-9 PMC1613187

[48] Maday S, Wallace KE, Holzbaur EL (2012) Autophagosomes initiate distally and mature during transport toward the cell soma in primary neurons. J Cell Biol 196: 407–417. 10.1083/jcb.201106120 22331844 PMC3283992

[49] Lee S, Sato Y, Nixon RA (2011) Lysosomal proteolysis inhibition selectively disrupts axonal transport of degradative organelles and causes an Alzheimer's-like axonal dystrophy. J Neurosci 31: 7817–7830. 10.1523/JNEUROSCI.6412-10.2011 21613495 PMC3351137

[50] Friedman LG, Lachenmayer ML, Wang J, He L, Poulose SM, et al (2012) Disrupted autophagy leads to dopaminergic axon and dendrite degeneration and promotes presynaptic accumulation of alpha-synuclein and LRRK2 in the brain. J Neurosci 32: 7585–7593. 10.1523/JNEUROSCI.5809-11.2012 22649237 PMC3382107

[51] Orimo S, Uchihara T, Nakamura A, Mori F, Kakita A, et al (2008) Axonal alpha-synuclein aggregates herald centripetal degeneration of cardiac sympathetic nerve in Parkinson's disease. Brain 131: 642–650. 18079166 10.1093/brain/awm302

[52] Anheim M, Elbaz A, Lesage S, Durr A, Condroyer C, et al (2012) Penetrance of Parkinson disease in glucocerebrosidase gene mutation carriers. Neurology 78: 417–420. 10.1212/WNL.0b013e318245f476 22282650

[53] Dauer W, Kholodilov N, Vila M, Trillat AC, Goodchild R, et al (2002) Resistance of alpha-synuclein null mice to the parkinsonian neurotoxin MPTP. Proc Natl Acad Sci U S A 99: 14524–14529. 12376616 10.1073/pnas.172514599 PMC137916

[54] Paine SM, Anderson G, Bedford K, Lawler K, Mayer RJ, et al (2013) Pale body-like inclusion formation and neurodegeneration following depletion of 26S proteasomes in mouse brain neurones are independent of alpha-synuclein. PLoS One 8: e54711 10.1371/journal.pone.0054711 23382946 PMC3559752

[55] Ochiai H, Sakamoto N, Suzuki K, Akasaka K, Yamamoto T (2008) The Ars insulator facilitates I-SceI meganuclease-mediated transgenesis in the sea urchin embryo. Dev Dyn 237: 2475–2482. 10.1002/dvdy.21690 18729225

[56] Takagi H, Inai Y, Watanabe S, Tatemoto S, Yajima M, et al (2012) Nucleosome exclusion from the interspecies-conserved central AT-rich region of the Ars insulator. J Biochem 151: 75–87. 10.1093/jb/mvr118 21930654

[57] Emelyanov A, Gao Y, Naqvi NI, Parinov S (2006) Trans-kingdom transposition of the maize dissociation element. Genetics 174: 1095–1104. 16951067 10.1534/genetics.106.061184 PMC1667081

[58] Ansai S, Inohaya K, Yoshiura Y, Schartl M, Uemura N, et al (2014) Design, evaluation, and screening methods for efficient targeted mutagenesis with transcription activator-like effector nucleases in medaka. Dev Growth Differ 56: 98–107. 10.1111/dgd.12104 24286287

[59] Bielawski J, Pierce JS, Snider J, Rembiesa B, Szulc ZM, et al (2010) Sphingolipid analysis by high performance liquid chromatography-tandem mass spectrometry (HPLC-MS/MS). Adv Exp Med Biol 688: 46–59. 20919645 10.1007/978-1-4419-6741-1_3

[60] Matsui H, Taniguchi Y, Inoue H, Kobayashi Y, Sakaki Y, et al (2010) Loss of PINK1 in medaka fish (Oryzias latipes) causes late-onset decrease in spontaneous movement. Neurosci Res 66: 151–161. 10.1016/j.neures.2009.10.010 19895857

[61] Lee BR, Kamitani T (2011) Improved immunodetection of endogenous alpha-synuclein. PLoS One 6: e23939 10.1371/journal.pone.0023939 21886844 PMC3158774

[62] Koike M, Nakanishi H, Saftig P, Ezaki J, Isahara K, et al (2000) Cathepsin D deficiency induces lysosomal storage with ceroid lipofuscin in mouse CNS neurons. J Neurosci 20: 6898–6906. 10995834 10.1523/JNEUROSCI.20-18-06898.2000 PMC6772823

