# Supplementary figures and images for: Viable Neuronopathic Gaucher Disease Model in Medaka (Oryzias latipes) Displays Axonal Accumulation of Alpha-Synuclein
Source: PLoS Genet. 2015 Apr 2;11(4):e1005065. doi: 10.1371/journal.pgen.1005065 (PMC4383526; doi:10.1371/journal.pgen.1005065)

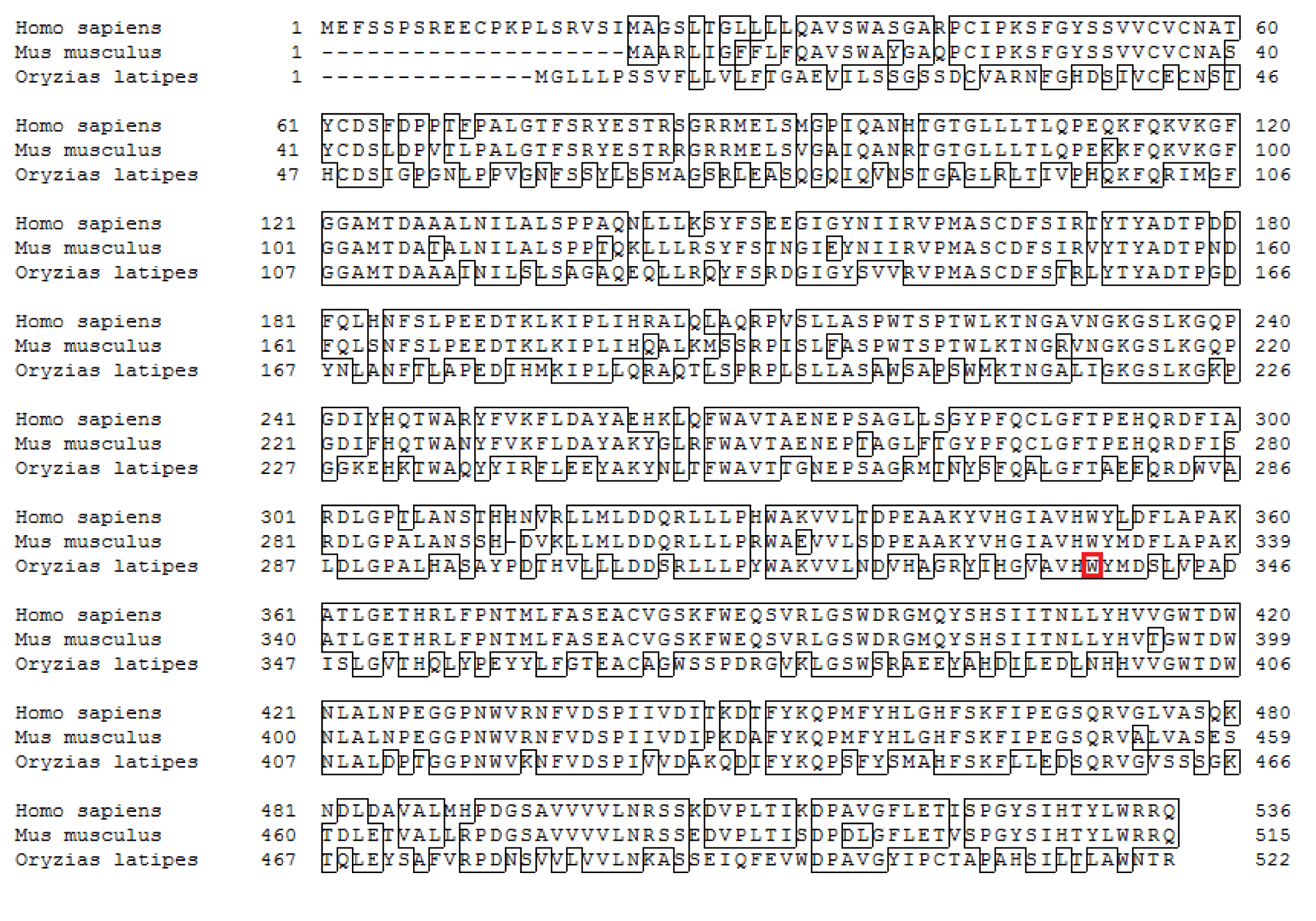

Supplement: S1 Fig — Sequence alignment of human (Homo sapiens), mouse (Mus musculus), and medaka (Oryzias latipes) GBA protein. Amino acids conserved in two or three species are outlined. The red outlining indicates the W337X mutation in the GBA nonsense mutant medaka. (TIF) [file pgen.1005065.s001.tif]

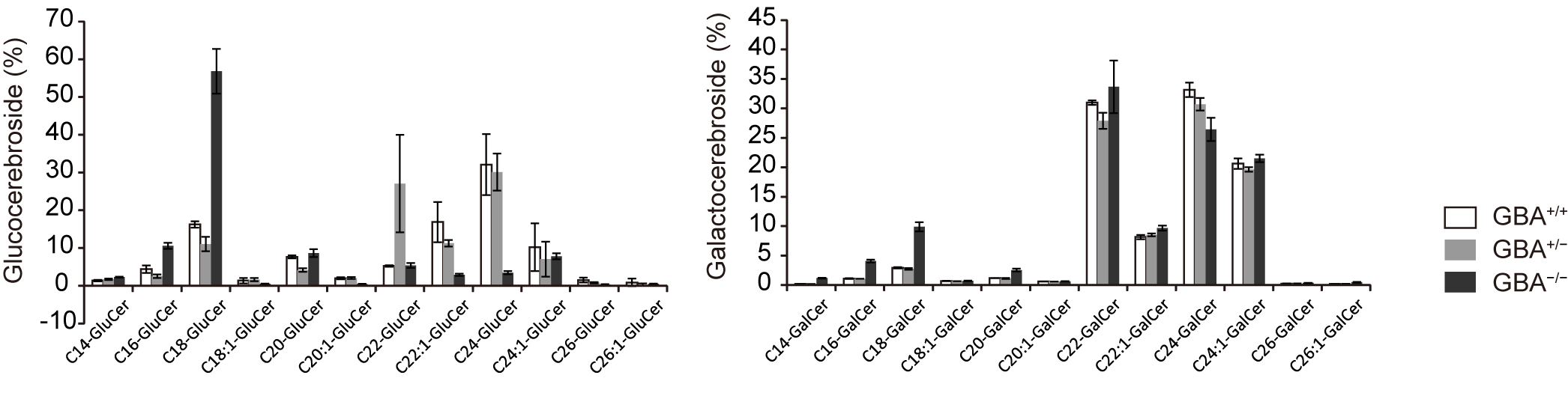

Supplement: S2 Fig — N-acyl chain distribution of glucocerebroside and galactocerebroside in medaka brains at 3 months (n = 3–4). For all analyses, data are the mean ± SEM. (TIF) [file pgen.1005065.s002.tif]

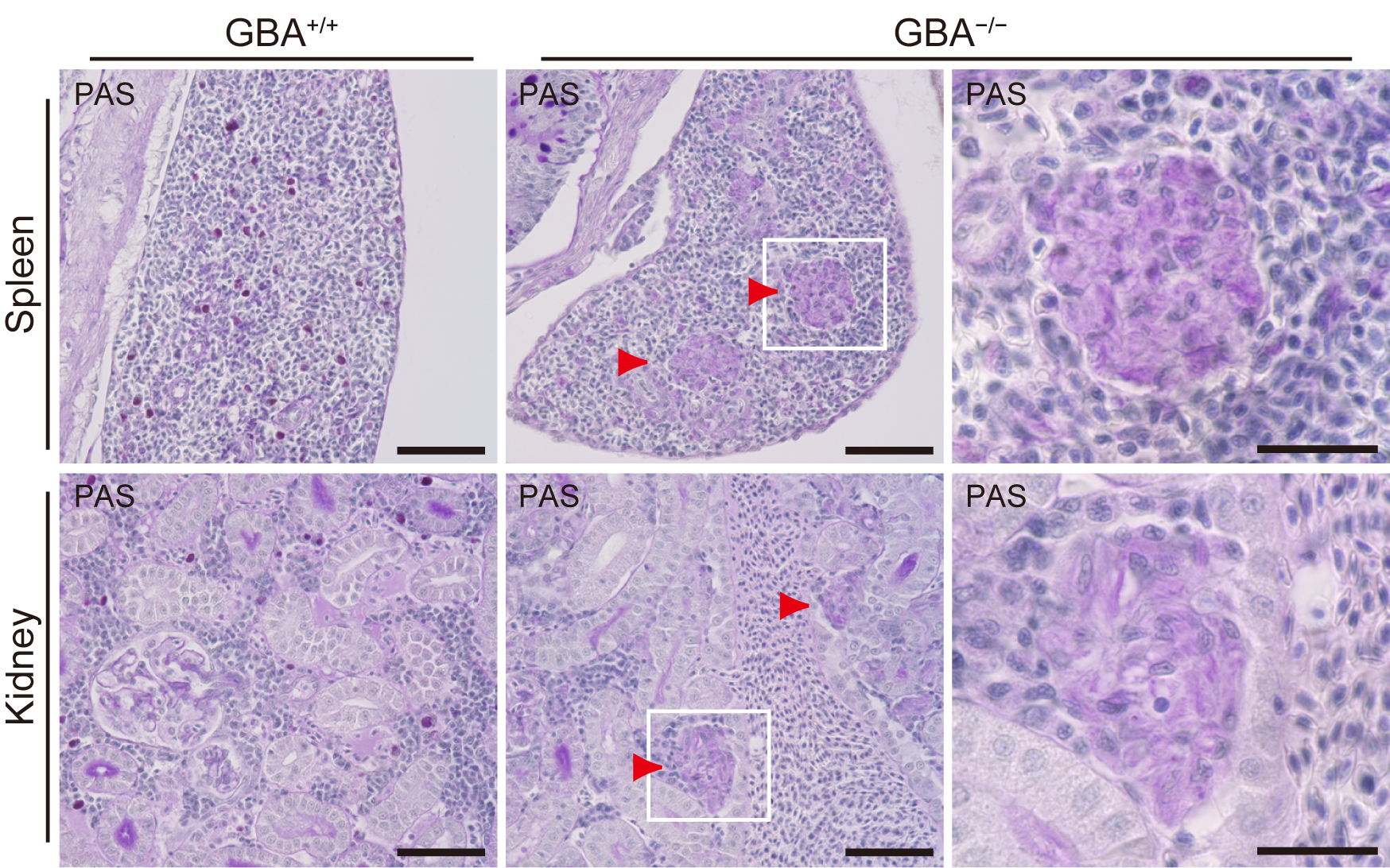

Supplement: S3 Fig — PAS staining of medaka spleen and kidney at 3 months. Clusters of abnormal PAS-positive cells in the spleen and kidney (arrowheads) and high-magnification images (right panels). Scale bars of right panels, 20 μm. Other scale bars, 50 μm. (TIF) [file pgen.1005065.s003.tif]

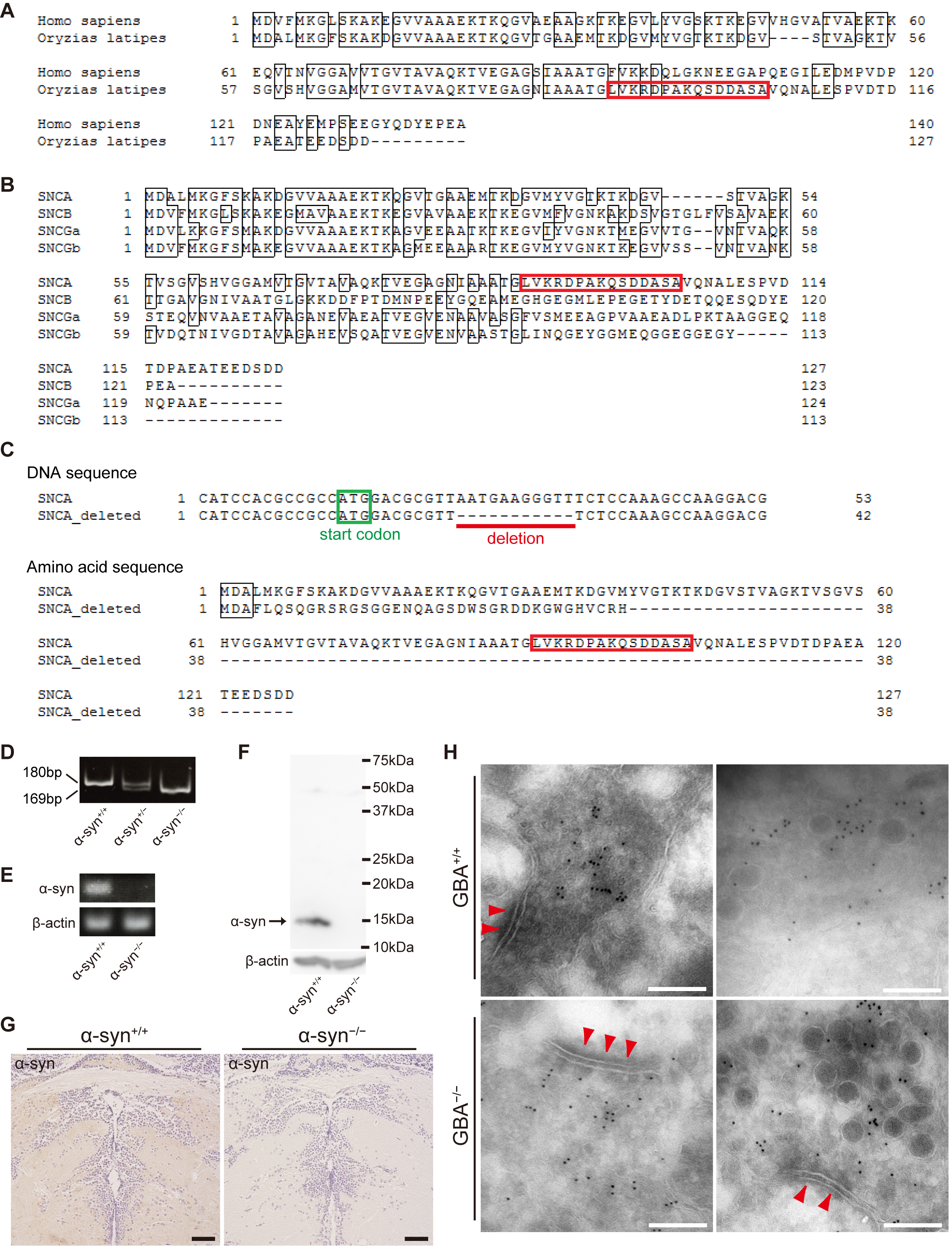

Supplement: S4 Fig — (A) Sequence alignment of human and medaka α-syn protein. Conserved amino acids are outlined. The red outlining indicates the epitope of medaka α-syn antibody. (B) Sequence alignment of medaka α-synuclein (SNCA), β-synuclein (SNCB), γ-synuclein-a (SNCGa), and γ-synuclein-b (SNCGb). Amino acids conserved in three or four proteins are outlined. The red outlining indicates the epitope of the medaka α-syn antibody. (C) Upper panel: DNA sequences of wild-type α-syn and deleted α-syn. Lower panel: Sequence alignment of wild-type α-syn and mutated α-syn. The red outlining indicates the epitope of the medaka α-syn antibody. (D) Polyacrylamide gel electrophoresis (PAGE) image of PCR for α-syn. PCR primers were designed to span the deleted region of α-syn, allowing the genotypes to be distinguished with PAGE. (E) RT-PCR for α-syn mRNA. One primer was designed to overlap the deleted region of α-syn. Intact α-syn mRNA was not detected in α-syn -/- medaka. (F) Western blot analysis of α-syn and β-actin. A 14-kDa putative α-syn band was observed only in α-syn +/+ medaka, suggesting the authenticity of the medaka α-syn antibody. (G) Immunohistochemistry with medaka α-syn antibody. α-syn immunostaining was observed only in α-syn +/+ medaka. Scale bars, 50 μm. (H) Immunoelectron micrograph of a presynaptic region with immunogold-labeled α-syn. Left panels: presynaptic area with small synaptic vesicles. Right panels: presynaptic area with large synaptic vesicles. The postsynaptic density is visible (arrowheads). Scale bars, 200 nm. (TIF) [file pgen.1005065.s004.tif]

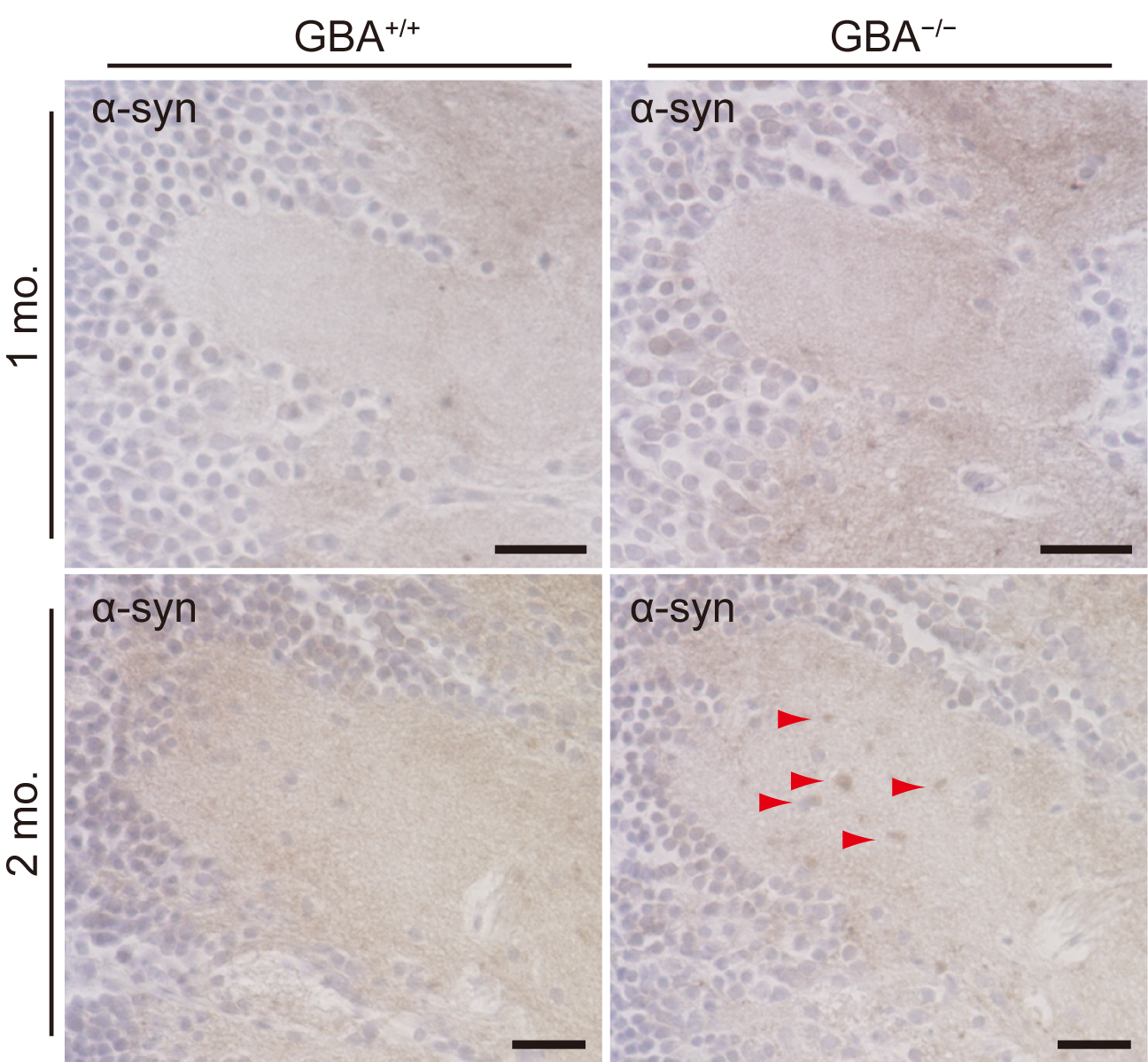

Supplement: S5 Fig — α-syn immunohistochemistry at 1 and 2 months after fertilization. α-syn accumulation was observed in GBA -/- medaka at 2 months (arrowheads). Scale bars, 20 μm. (TIF) [file pgen.1005065.s005.tif]

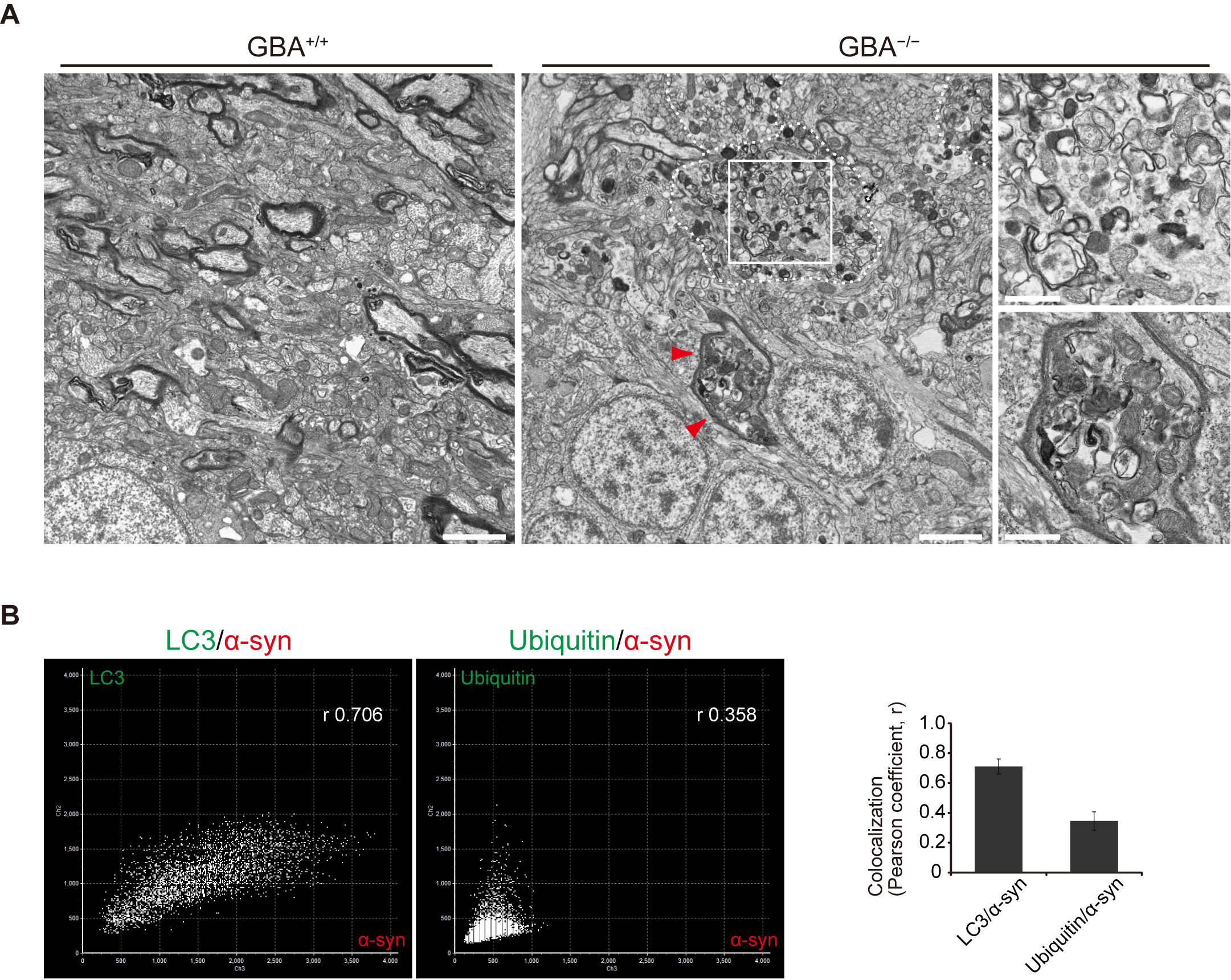

Supplement: S6 Fig — (A) Transmission electron micrographs of neuropil. Left and middle panels: Neuropil of a GBA +/+ and a GBA -/- medaka, respectively. Swellings of both myelinated (arrowheads) and unmyelinated (outlined by dashed lines) axons were found in GBA -/- medaka, which contain vacuoles and electron-dense bodies. Scale bars, 2 μm. Right panels: High-magnification images of swellings of myelinated and unmyelinated axons (lower and upper panels, respectively). Scale bars, 500 nm. (B) Co-localization analysis for different markers in axonal swellings of GBA -/- medaka. The correlation between LC3, ubiquitin, and α-syn signals are shown as the pixel scatter diagrams and a graph (n = 6). For all analyses, data are the mean ± SEM. (TIF) [file pgen.1005065.s006.tif]

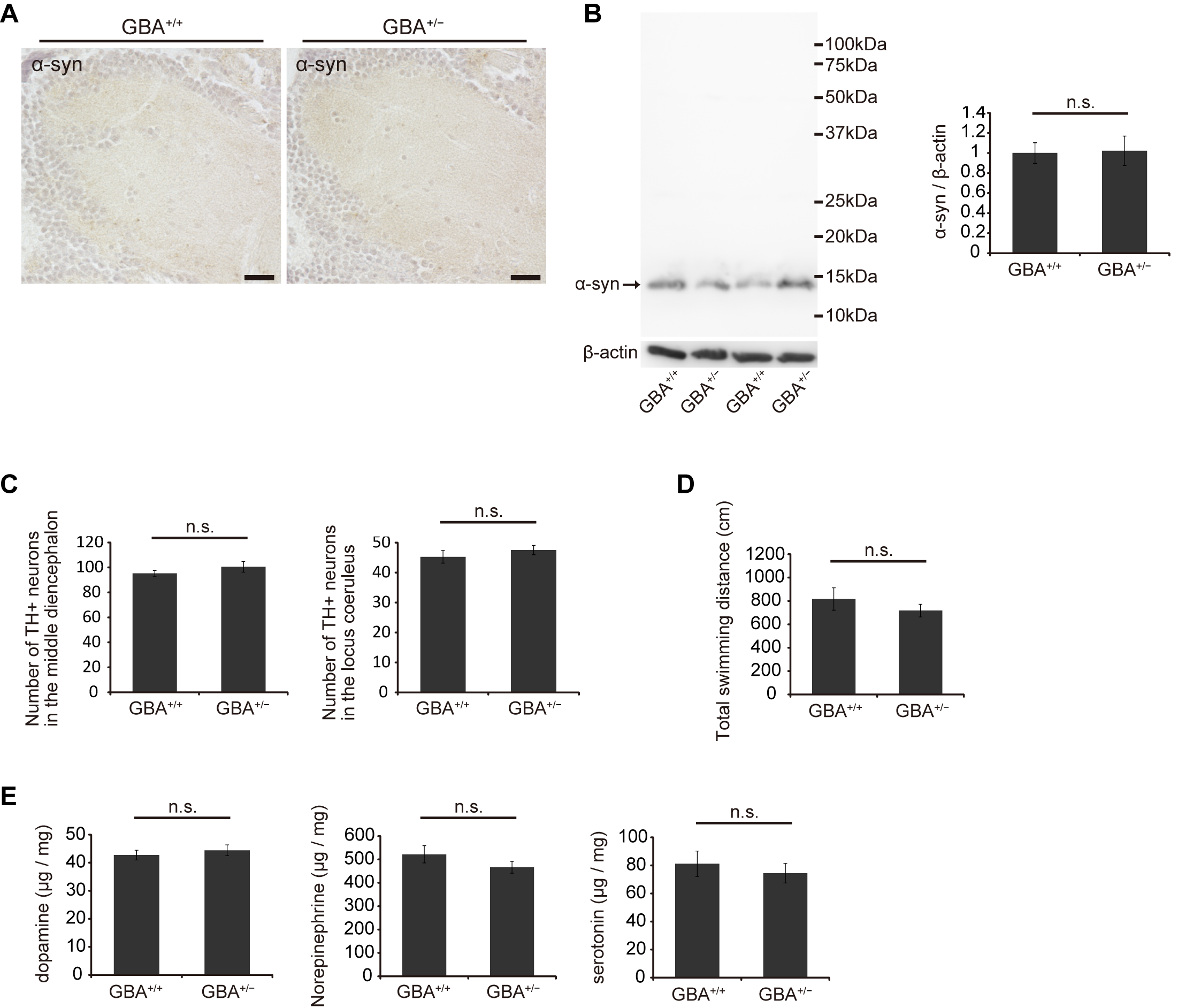

Supplement: S7 Fig — (A) α-syn immunohistochemistry at 12 months. α-syn accumulation was not observed in GBA +/- medaka. Scale bars, 20 μm. (B) Western blot analysis of α-syn and β-actin (n = 5–6). (C) Numbers of TH-positive neurons in the middle diencephalon and TH-positive neurons in the locus coeruleus at 12 months (n = 4). (D) Total swimming distance at 12 months (n = 12). (E) Amounts of dopamine, noradrenaline, and serotonin in the brains at 12 months measured with high performance liquid chromatography. All values are expressed as the amount (μg) per protein (mg) (n = 12). For all analyses, data are the mean ± SEM. A two-tailed paired Student’s t-test was used to determine the statistical significance. (TIF) [file pgen.1005065.s007.tif]

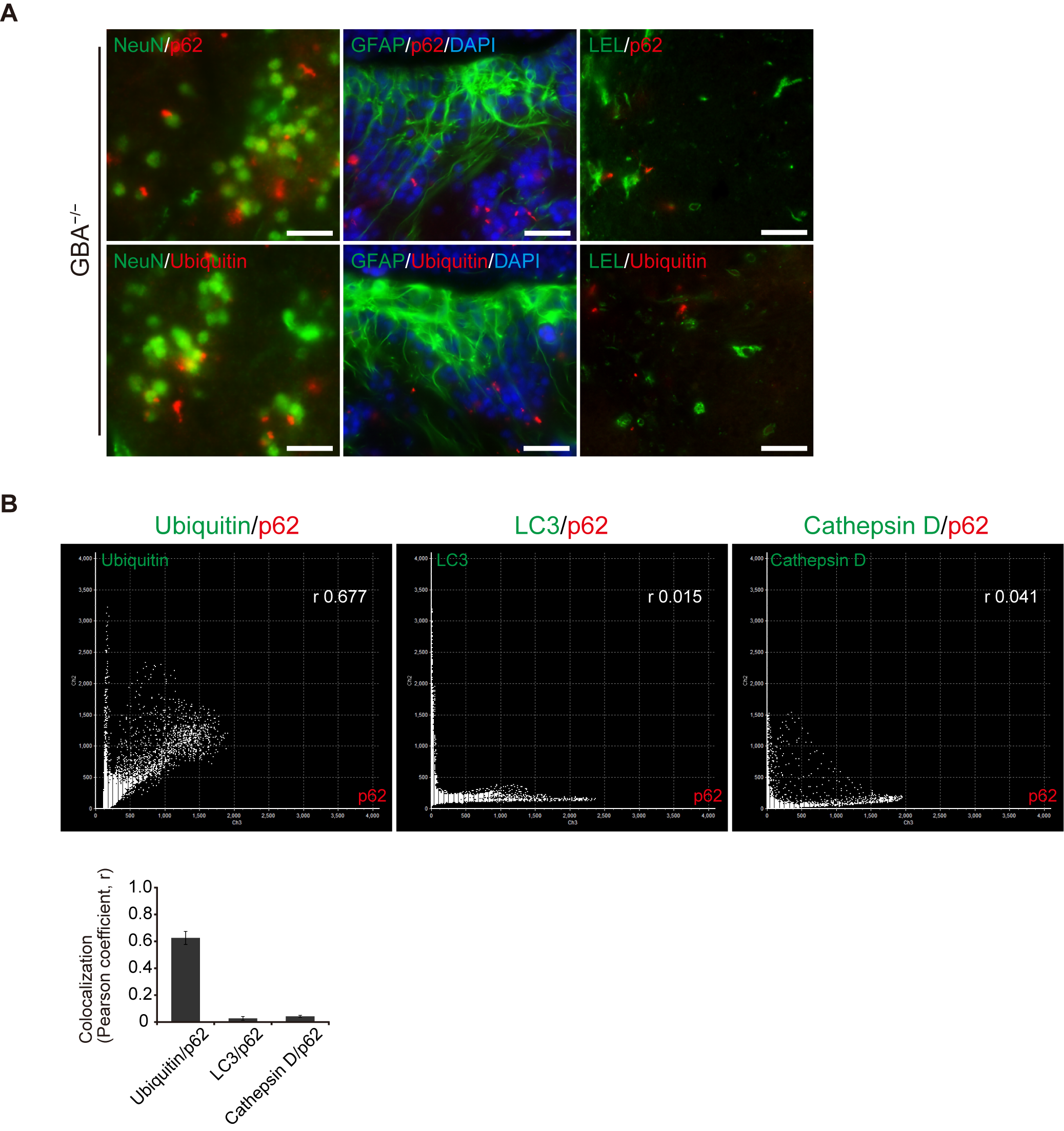

Supplement: S8 Fig — (A) Double immunostaining in GBA -/- medaka at 3 months. Upper panels: NeuN (green), GFAP (green), or LEL (green) and p62 (red). Lower panels: NeuN (green), GFAP (green), or LEL (green) and ubiquitin (red). Nuclei were visualized with DAPI (blue). p62- and Ubiquitin-positive aggregates were localized only in NeuN-positive neurons, but not in GFAP-positive radial glial cells or LEL-positive microglia. Scale bars, 20 μm. (B) Co-localization analysis for different markers in the brains of GBA -/- medaka. The correlation between LC3, ubiquitin, and α-syn signals are shown as the pixel scatter diagrams and a graph (n = 6). For all analyses, data are the mean ± SEM. (TIF) [file pgen.1005065.s008.tif]

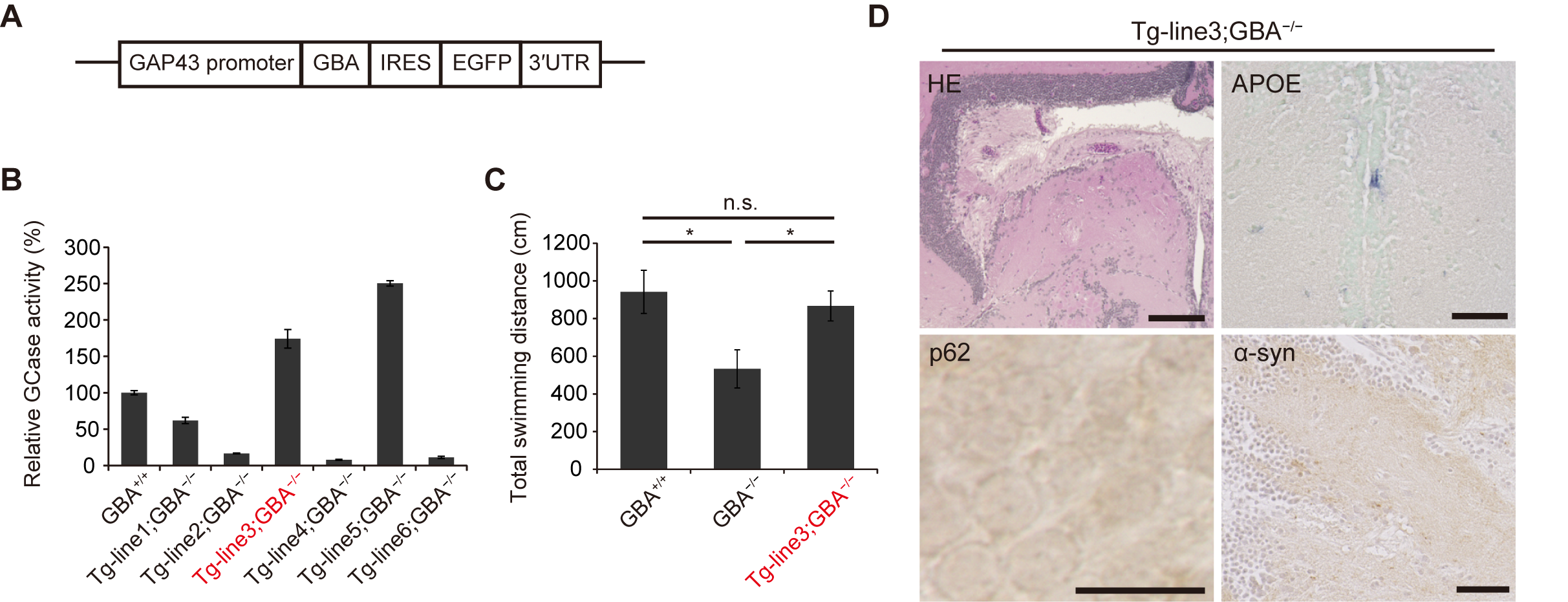

Supplement: S9 Fig — (A) Transgenic construct used to establish GBA transgenic medaka. (B) GCase activity in the brains of each Tg(GAP-43:GBA);GBA -/- lines (described as Tg-line No.;GBA -/- in the figure) at 3 months (n = 5–6). (C) Total swimming distance during 3 min in GBA +/+, GBA -/-, and Tg(GAP-43:GBA)line3;GBA -/- medaka (n = 8, *p < 0.05). (D) Hematoxylin and eosin staining, APOE in situ hybridization, and p62 and α-syn immunohistochemistry of Tg(GAP-43:GBA)line3;GBA -/- medaka showed no major abnormalities. Scale bars, 100 μm, 50 μm, 10 μm, 20 μm, respectively. For all analyses, data are the mean ± SEM. (TIF) [file pgen.1005065.s009.tif]
